# Multi-omics reveals that dietary *Piper sarmentosum* Roxb. extract improves goat meat quality via the microbiota–muscle amino acid metabolic axis

**DOI:** 10.1186/s40104-026-01509-5

**Published:** 2026-10-13

**Authors:** Guodong Ren, Tingshuo Yang, Ziyang Sheng, Yixin Chen, Lexiang Chen, Yuting Wang, Peilin He, Hailing Luo

**Affiliations:** 1 Sanya Institute of China Agricultural University, Sanya, 572025 China; 2 State Key Laboratory of Animal Nutrition and Feeding, College of Animal Science and Technology, China Agricultural University, No.2 Yuanmingyuan West Road, Haidian, Beijing, 100193 China

**Keywords:** Goat, Meat quality, Metabolome, *Piper sarmentosum* Roxb. extract, Rumen microbiota

## Abstract

**Background:**

Improving meat quality through nutritional regulation is an important approach in ruminant production. Growing evidence suggests that rumen microbiota influences host metabolism via microbial metabolites, thereby affecting meat quality. However, whether *Piper sarmentosum* Roxb. extract (PSE), a medicinal and edible plant-derived extract, improves meat quality through rumen microbiota–host metabolic interactions remains unclear. Thirty-six weaned male Hainan black goats (approximately 3 months old) were randomly assigned to four dietary treatments supplemented with 0, 200, 400, or 600 mg/kg PSE (dry matter basis), respectively. After a 90-day feeding trial, goats were fasted for 12 h before slaughter, and rumen and *longissimus lumborum* muscle samples were collected for subsequent analyses.

**Results:**

Supplementation with 600 mg/kg PSE significantly enhanced meat quality traits, including higher ultimate pH, improved color stability, and increased water-holding capacity, without affecting growth performance or carcass traits. Transcriptomic and metabolomic analyses revealed that PSE supplementation was associated with remodeling of amino acid and energy metabolism in muscle, particularly glutamate-related pathways. These metabolic changes were accompanied by increased expression of key metabolic genes, including *BCAT2*, *IDH1*, and *IDH3A*, alongside alterations in energy-related metabolites, such as the downregulation of carnitine and upregulation of creatinine. Concomitant shifts in rumen microbial composition were observed, with PSE supplementation increasing the abundance of genera like *Weissella* and *Ruminococcus*, while reducing potentially opportunistic genera, such as *Acinetobacter* and *Klebsiella*. *Weissella* showed strong positive correlations with elevated rumen metabolites like serine, threonine, choline, and hypoxanthine. Metagenome-assembled genome analysis of *Weissella cibaria* revealed an enrichment of genes related to amino acid metabolism and glycosyltransferase families. Correlation and mediation analyses further revealed associations among *Weissella* abundance, rumen metabolites, and muscle metabolic traits, supporting a potential microbiota–metabolite–host metabolic relationship.

**Conclusions:**

Collectively, this study provides new insights into the association network linking PSE-induced alterations in rumen microbiota, microbial metabolites, and host muscle metabolism in relation to goat meat quality. These findings suggest that PSE supplementation may improve meat quality through coordinated changes in rumen microbial ecology and muscle metabolic profiles.

**Supplementary Information:**

The online version contains supplementary material available at https://doi.org/10.1186/s40104-026-01509-5.

## Introduction

Meat from small ruminants, including goats and sheep, is a globally consumed source of high-quality dietary protein and is highly valued for its nutritional composition and distinctive sensory properties. Goats, particularly indigenous breeds, play an important role in food security and rural livelihoods in tropical and subtropical regions because of their adaptability to diverse environmental conditions and ability to utilize low-quality feed resources [1]. Therefore, improving production efficiency and meat quality through effective nutritional strategies is of considerable importance for enhancing both the economic value of goat production and consumer satisfaction. As a critical determinant of both consumer acceptance and economic value, the quality of meat from sheep or goat is largely shaped by postmortem muscle metabolism, which governs essential traits such as pH decline, color stability, and water-holding capacity [2, 3]. These biochemical processes are strongly influenced by preslaughter muscle metabolic status, which is affected by genetic background, sex, and nutritional regulation [4]. Compared with relatively stable genetic backgrounds, nutritional regulation offers greater plasticity and interventional potential for improving meat quality.

The gut microbiota is increasingly recognized as a key regulator of host metabolism, primarily through the production of a wide range of metabolites that function as signaling molecules modulating host metabolic pathways [5, 6]. Rumen-derived metabolites, including short-chain fatty acids, microbial amino acid-related compounds, and other bioactive metabolites, can be absorbed through the rumen epithelium and enter the host circulation, thereby contributing to systemic metabolism and nutrient utilization in peripheral tissues [7]. Recent studies have highlighted the importance of microbiota–host interactions in regulating animal growth and metabolic homeostasis [8, 9]. Among these, skeletal muscle responds to circulating nutrients and metabolites by modulating energy and amino acid metabolism, thereby influencing postmortem metabolism and meat quality [10]. Nevertheless, whether rumen microbial metabolism contributes to muscle metabolic remodeling and subsequently influences meat quality remains largely unclear.

Given the increasing demand for high-quality meat from sheep or goat and sustainable small-ruminant production, nutritional interventions have attracted considerable attention as an effective strategy for improving meat quality and production efficiency [11]. Among these, natural plant-derived bioactive compounds have been widely investigated as dietary strategies to improve meat quality [12, 13]. *Piper sarmentosum* Roxb., a traditional plant of medicine and food homology widely distributed in tropical and subtropical regions of Asia [14–16], contains abundant flavonoids and essential oils with reported antioxidant and metabolic regulatory properties [17–20], suggesting its potential as a dietary phytogenic additive for modulating animal metabolism. However, whether *Piper sarmentosum* Roxb. extract (PSE) can improve ruminant meat quality through alterations in rumen microbial composition and microbiota-associated host metabolism remains largely unexplored. The functional roles of key microbial taxa involved in this potential rumen–muscle metabolic axis are also poorly characterized.

Therefore, this study aimed to investigate whether dietary PSE supplementation affects meat quality in Hainan black goats through the rumen microbiota–metabolite–muscle axis. We integrated rumen metagenomics and metabolomics with muscle metabolomics and transcriptomics to characterize the associations among PSE-induced changes in rumen microbial communities, microbial metabolites, muscle metabolism, and meat quality. We hypothesized that PSE supplementation would improve meat quality by modulating rumen microbial metabolism and its subsequent effects on host muscle metabolism. This study will provide a new insight into the potential associations between rumen microbiota, microbial metabolites, and host muscle metabolism, and proposes a microbiota–metabolite–muscle interaction framework underlying the effects of dietary PSE supplementation.

## Materials and methods

### Animals, experimental design and sampling

The experimental protocols for the Hainan black goats were approved by the China Agricultural University Animal Care and Use Committee, and the animal feeding and slaughter procedures followed the university's guidelines (Beijing, China; approval No. AW20206202-1-12). Thirty-six male Hainan black goat kids of the same age (approximately 3 months old) and with similar initial body weight (IBW, 9.5 ± 0.25 kg) were selected and fed up to 6 months of age at the Hainan Black Goat Breeding Base in Danzhou city (109°51′E, 19°52′N), Hainan Province, China. All goats belonged to the same breeding farm and originated from different sires, but were raised under identical management conditions. The goats were randomly assigned to four dietary treatment groups using the RAND function in Microsoft Excel (2019), with 9 goats per group (*n* = 9). The goats were fed one of the four diets: basal diet (Table S1) supplemented with either 0 (control, CON), 200 (200PSE), 400 (400PSE) or 600 (600PSE) mg/kg PSE (dry matter basis). The PSE supplementation levels were selected based on previous studies [20] and our preliminary in vitro rumen fermentation results [21]. PSE was provided by the Tropical Crops Genetic Resources Institute, Chinese Academy of Tropical Agricultural Sciences. The leaves and stems of *Piper sarmentosum* Roxb. were collected from the Tropical Botanical Garden in Danzhou, Hainan province, China. The plant material was subsequently pulverized and extracted with 95% ethanol using a cold maceration method. The extract was concentrated by rotary evaporation to obtain the crude extract, which was subsequently freeze-dried, mixed with zeolite powder at a 1:1 (w/w) ratio, and stored in sealed dark bottles at 4 °C until use. The content of bioactive compounds in PSE was supplemented in Table S2. All goats had ad libitum access to the basal diet and drinking water. The daily PSE dose for each goat was thoroughly mixed with a small amount of concentrate and offered individually before the morning feeding. After the PSE-containing concentrate was consumed, the remaining basal diet was provided ad libitum. The experimental trial lasted 105 d, with 15 d for adaptation and 90 d for data collection. Each goat was individually identified and dewormed before being housed in an individual pen throughout the entire experimental period and fed twice daily. The body weights of the animals were recorded before the morning feeding on the first day and on d 90 (final body weight, FBW). The amount of feed offered to each goat was recorded daily, and feed refusals were collected and weighed before the morning feeding on the following day. The daily dry matter intake (DMI) of all goats was recorded every day. The average daily gain (ADG), average daily feed intake (ADFI) and feed-to-gain ratio (F/G) of all goats were calculated at the end of trial.

At the end of the feeding period, all goats were slaughtered after being deprived of feed and water for 12 h. The slaughter procedure was following our previous study [12]. Slaughter performance was evaluated in this study, including live weight, head weight, hooves weight, fur weight, carcass weight, dressing percentage, bone weight, net meat weight, pure meat percentage, eye muscle area, and GR value. The right *longissimus lumborum* muscle was collected to determine pH, meat color, cooking loss, drip loss, and the left was determined to meat nutritional composition. Meanwhile, rumen contents and *longissimus lumborum* muscle samples were collected from all goats (*n* = 9 per treatment group), immediately placed in liquid nitrogen, and stored at −80 °C for further analysis.

### Meat quality analysis

The *longissimus lumborum* muscle samples between 6^th^ and 12^th^ thoracic vertebrae from all goats were collected in plastic bags numbered kept at 4 °C. After calibrated using pH 4.0 and 7.0 buffers, a digital Testo 205 pH meter with automatic temperature compensation (Testo SE & Co. KGaA, Shanghai, China) was used for muscle pH determination. Each muscle was assessed through an incision made in the muscle in the shape of a cross at 45 min and 24 h postmortem. Meat color indices, including lightness (L*), redness (a*), and yellowness (b*), were carried out with the aid of a high-quality spectrophotometer NS800 (Φ8 mm aperture, 10° standard observer, D65 illuminant; 3NH Technology Co., Ltd., Shenzhen, China) on fresh cuts. After 30 min of blooming at environmental temperature (approximately at 25 °C), three different planes were measured at 45 min and 24 h postmortem, and their mean was used for subsequent analysis.

Following the removal of external adipose tissue and connective tissue, the individual muscle samples between 6^th^ and 12^th^ thoracic vertebrae were weighed and cooked in a water bath at 75 °C for 35 min until its core temperature increased at 70 °C finally. When cooled to room temperature, the samples were blotted dry on filter paper and reweighed. The final value of cooking loss was calculated according to Geesink et al. [22]. Drip loss was also determined to use previous method described by Liu et al. [23] and Wang et al. [24]. The meat nutritional composition including crude ash, crude protein (CP) and ether extract (EE) were determined following Association of Official Agricultural Chemists procedures [25]. The nitrogen content of the samples was measured using a fully automated Kjeldahl apparatus (Kjeltec 8400, fossanalytics Co., Ltd., Beijing, China).

### Non-targeted/targeted metabolomic analysis

Based on the results of meat quality, the CON and 600PSE groups showing the greatest differences were selected for further analysis. Rumen contents or *longissimus lumborum* muscle samples (100 mg) was ground under liquid nitrogen freezing, and then transferred to resuspension solution containing 500 μL of pre-cooled methanol (80%) and vortex-mixed thoroughly. Subsequently, the mixed samples were placed on ice for a 5-min incubation period and centrifuged at 15,000 × *g* at 4 °C for 20 min. The resulting supernatants were adjusted to a concentration of 53% methanol by dilution with LC–MS-grade water, followed by a second centrifugation at 15,000 × *g* at 4 °C for 20 min. The final supernatants were retained via filtration through a 0.22-μm membrane for further analysis.

The raw data were collected and analyzed via a Vanquish UHPLC system (Thermo Fisher, Germany) interconnected for use with an Orbitrap Q Exactive™ HF-X mass spectrometer (Thermo Fisher, Germany). A chromatographic column of Hypersil GOLD (100 mm × 2.1 mm, 1.9 µm) was utilized for the separation process. The gradient program was set for 17 min at a consistent flow rate of 0.2 mL/min. In positive mode, the mobile phases consisted of eluent A, which was 0.1% formic acid in water, and eluent B, which was methanol. For negative mode, eluent A was 50 mmol/L ammonium acetate at a pH of 9.0, and eluent B was methanol. The solvent gradient was programmed as follows: 2% B for 1.5 min; 2%–100% B over 10.5 min; 100% B for 2.0 min; 100%–2% B over 0.1 min; and 2% B for 2.9 min. An Q Exactive™ HF-X mass spectrometer in positive/negative polarity mode has a scanning range of *m/z* 100–15,00 and it has a data-dependent (MS/MS) secondary scanning mode. The following parameters of ESI source were shown: a spray voltage of 3.5 kV, a sheath gas flow rate of 35 psi, an auxiliary gas flow rate of 10 L/min, an ion transfer tube temperature of 320 °C, an S-lens RF level of 60, and an auxiliary gas heater temperature of 350 °C. Using XCMS to align peaks, pick peaks, and quantify each metabolite in the raw data files obtained from UHPLC-MS/MS. Then, the metabolite peak identification was based on the set 10 ppm mas matching deviation and adduct ion, and it was compared with a high-quality secondary spectrum database (mzCloud (https://www.mzcloud.org/), mzVault, and Mass List databases).

After data preprocessing with Pareto scaling, (O)PLS-DA was performed to explore metabolites differences. The VIP scores from the (O)PLS-DA were extracted to identify key contributors. The following threshold was used to identify differentially expressed metabolites (DMs): VIP score > 1, *P*-value < 0.05 based on Student's *t*-test, and fold-change value > 1 or < 1. Fold-change values for each metabolite were calculated by comparing the mean abundance between the CON and 600PSE treatment groups (CON vs. 600PSE). Two databases were used to further annotate and validate the DMs, including the Kyoto Encyclopedia of Genes and Genomes (KEGG, https://www.kegg.jp/) and the Human Metabolome Database (HMDB). KEGG pathway enrichment analysis was then performed based on the DMs. A hypergeometric test was performed to evaluate the statistical significance of the enriched pathways.

The amino acid content of *longissimus lumborum* muscle was measured using a high-speed amino acid analyzer (LA8080, Hitachi, Ltd., Japan), following the previously reported method of Chai et al. [26]. Fresh muscle samples were homogenized with 10 mL of 0.01 mol/L hydrochloric acid, thoroughly mixed with three drops of phenol, and then hydrolyzed at 110 ± 1 °C in a thermostat for 22 h. The filtrate (1 mL) was transferred to a 15-mL test tube after filtration, dried under nitrogen, and subsequently mixed with 1 mL of sodium citrate buffer solution (pH = 2.2). The mixture was then filtered using a 0.22-μm pore-size membrane filter. The analysis was performed using the resulting supernatant. Amino acid contents were expressed as g/100 g of meat. 

### Transcriptome analysis and qPCR validation

The *longissimus lumborum* muscle samples preserved in liquid nitrogen were used for total RNA extraction. Total RNA was extracted using TRIzol reagent kit (Invitrogen, Carlsbad, CA, USA) according to the manufacturer’s protocol. RNA quality was assessed on an Agilent 2100 Bioanalyzer (Agilent Technologies, Palo Alto, CA, USA) and checked using RNase free agarose gel electrophoresis. After total RNA was extracted, eukaryotic mRNA was enriched by Oligo(dT) beads. Then the enriched mRNA was fragmented into short fragments using fragmentation buffer and reversely transcribed into cDNA by using NEBNext Ultra RNA Library Prep Kit for Illumina (NEB #7530, New England Biolabs, Ipswich, MA, USA). The purified double-stranded cDNA fragments were end repaired, A base added, and ligated to Illumina sequencing adapters. The ligation reaction was purified with the AMPure XP Beads (1.0X) and amplified with polymerase chain reaction (PCR). The resulting cDNA library was sequenced using Illumina Novaseq 6000 by Gene Denovo Biotechnology Co., Ltd. (Guangzhou, China).

Subsequent to the acquisition of raw reads from the sequencing platform, a filtration process was implemented using the fastp software version 0.18.0 [27]. The short-reads alignment tool Bowtie2 (version 2.2.8) [28] was used to map reads to the ribosomal RNA (rRNA) database. The remaining clean reads were further used in assembly and gene abundance calculation. An index of the reference genome was built, and paired-end clean reads were mapped to the *Capra hircus* reference genome (Ensembl_release109) using HISAT2 (v2.1.0) [29]. The mapped reads of each sample were assembled by using StringTie v1.3.1. For each transcription region, a TPM (Transcripts Per Kilobase of exon model per Million mapped reads) value was calculated to quantify its expression abundance and variations, using RSEM [30] software. RNAs differential expression analysis was performed by DESeq2 software [31] between two different groups. Genes/transcripts were designated as differentially expressed genes/transcripts (DEGs) if they met the following criteria: a false discovery rate (FDR) of less than 0.05 and an absolute fold change of over 1.5 or less than 1.5. A threshold of FDR ≤ 0.05 was used to map all DEGs into the KEGG pathway enrichment analysis. Gene set enrichment analysis (GSEA) was used to perform differential KEGG analysis in the CON and 600PSE group, with a screening threshold of |Normalized enrichment score, NES| > 1 and FDR < 0.05. To validate the transcriptome data, quantitative real-time polymerase chain reaction (qPCR) was performed on key differentially expressed genes (*GLUL*, *IDH1*, *CAMK2A*, *PDXK*, *FADS1*, *SCD*, and *NRG4*) identified in the RNA-seq analysis. Specifically, the procedures and details were supplemented to Table S8, S9, and Fig. S1.

### Metagenomic sequencing and binning analysis

High‐quality genomic DNA from rumen contents was extracted using HiPure Bacterial DNA Kits (Magen, Guangzhou, China) according to the manufacturer's instructions. Qualified metagenomic DNA with an insert size of 350 bp was firstly fragmented, and then end-repaired, A-tailed, and adaptor ligated by using NEBNext^®^ ΜLtra™ DNA Library Prep Kit for Illumina (NEB, USA) according to the preparation protocol. After purified and quantified, genome sequencing was performed on the Illumina Novaseq 6000 sequencer using the pair-end technology (PE150). Raw data from Illumina platform were filtered using FASTP (v0.18.0): (a) removing reads with 10% unidentified nucleotides (N); (b) removing reads with ≥ 50% bases having phred quality scores ≤ 20; (c) removing reads aligned to the barcode adapter. After filtering, the resulted clean reads were used for genome assembly. After filtering, clean reads of each sample were assembled individually using MEGAHIT (v1.1.2) [32]. Genes were predicted based on the final assembly contigs (> 500 bp) using MetaGeneMark (version 3.38) [33]. The predicted genes ≥ 300 bp in length from all samples were pooled and combined based on ≥ 95% identity and 90% reads coverage using CD-HIT (v4.6) [34] in order to reduce the number of redundant genes for the downstream assembly step. The reads were re-aligned to the predicted genes using Bowtie (v2.2.5) [28] to count the number of reads. Final gene catalogue was obtained from non-redundant genes with gene reads count > 2. Using DIAMOND (v0.9.24) [35] to annotate the Unigenes by aligning them against databases such as the RefSeq non-redundant proteins database, the CAZy database (Carbohydrate-Active enZYmes Database), and the KEGG database. Using the MEGAN software (v6.19.9) [36] and the LCA (Lowest Common Ancestor) algorithm [37], the obtained unigene sequences were aligned against the Nr database (non-redundant protein sequences database) to obtain species annotations for the genes, and species abundance was determined based on gene abundance. Metagenomic alpha diversity indices, including Chao1, Shannon, and Simpson, were compared using Welch’s *t*-test. Beta diversity was assessed by principal coordinates analysis (PCoA) based on Bray–Curtis distance, and statistical significance was evaluated using analysis of similarities (ANOSIM). For functional profiles, beta diversity of KEGG and CAZy annotations was further analyzed using partial least squares discriminant analysis (PLS-DA). Linear discriminant analysis effect size (LEfSe) was applied to identify differentially abundant taxa between groups, with an LDA score threshold of > 2.

Using clean reads obtained from metagenomic sequencing, the quality-controlled clean reads were aligned to the host reference genome using Bowtie2 version 2.3.5.1 The filtered reads were assembled into contigs using MEGAHIT (v1.1.2) within the 27–127 k-mer range, and the resulting contigs were used for binning analysis. The metagenome assemblies (length > 1.5 kb) were binned using METABAT v2.12.1 [38]. The completeness and contamination of bins were evaluated using machine learning software CheckM2 v1.0.2 [39]. All bins were classified into high quality (completeness ≥ 80% and contamination ≤ 5%), medium quality (completeness ≥ 50% and contamination ≤ 10%), and low quality (completeness < 50%) based on genome reporting standards [40]. High quality and medium quality bins were retained for genome dereplication by dRep v3.4.2 [41]. The representative genomes selected by dRep could be considered as Species-level genomes or metagenome-assembled genomes (MAGs). The remaining MAGs were classified using GTDB (Genome Taxonomy Database, Release 220) [42] and GTDB-Tk v2.4.0 [43] with the ‘classify_wf’ workflow. The abundance of MAGs in each sample was calculated using the Quant_bins module of metaWRAP v1.3.2 [44], and subsequent species analysis was performed based on the MAG abundance and species annotation results. The Prokka software (v1.14.6) [45] was used to predict the coding genes of each MAG. Then, DIAMOND (v0.9.25) [35] was used to align the coding genes of each MAG with the KEGG and CAZy database, and the protein sequences with the highest similarity were selected to obtain the corresponding protein functional annotations.

### Statistical, correlation, and mediation analysis

Data for growth performance, slaughter performance, and meat quality trait analysis were described using Proc Mixed Procedure of SAS (SAS^®^ OnDemand for Academics, https://welcome.oda.sas.com/), with four treatments (CON, 200PSE, 400PSE and 600PSE) as a fixed effect and random terms for goats. The differences between the means were tested using Tukey’s multiple range test when significant differences were detected at *P* < 0.05. Statistical significance was defined at *P* < 0.05. An orthogonal polynomial SAS CONTRAST statement was used to test the linear and quadratic effects of four PSE levels.

Spearman’s correlation and Mantel test analyses were conducted using the Chiplot platform (https://www.chiplot.online/). Microbial abundance profiles were subjected to centered log-ratio (CLR) transformation to account for compositionality, whereas metabolite abundances were normalized by Z-score transformation before correlation analyses. MetOrigin 2.0 (https://metorigin.met-bioinformatics.cn/) was used to annotate the potential biological origins of differential rumen metabolites. Correlation networks illustrating the associations between differential microbial taxa and metabolites were constructed using Spearman’s correlation analysis (|*r*| > 0.7 and FDR < 0.05) and visualized using Gephi software (version 0.10.1).

The mediation analysis was conducted using SPSS software (version 26.0), and the mediation model was constructed using the PROCESS plugin (version 4.2) developed by Andrew F. Hayes. Significance tests were conducted using the Bootstrap method, with 5,000 bootstrap repetitions, to assess the significance of the statistical mediation effects and their confidence intervals. The significance of the mediating effects was determined based on the 95% confidence interval (CI). A mediating effect was considered significant if the confidence interval did not include 0. All statistical tests were two-tailed, with a significance level set at α = 0.05.

## Results

### Growth performance, slaughter performance, and meat quality

No significant differences were observed in IBW, FBW, or DMI among the treatment groups (*P* > 0.05; Table 1). However, dietary supplementation with 400 or 600 mg/kg PSE significantly decreased the feed-to-gain ratio (F/G) compared with the CON group, with a linear effect observed through treatments (*P* < 0.05). Average daily gain (ADG) tended to increase with PSE supplementation (*P* = 0.074).

**Table 1:** Effect of dietary supplementation with PSE on the growth performance of Hainan black goats

Items^2^	Groups^1^				SEM	*P*-value		
	CON	200PSE	400PSE	600PSE		Treatment	Linear	Quadratic
IBW, kg	9.11	9.23	9.38	8.92	0.253	0.961	0.892	0.676
FBW, kg	12.91	13.37	14.10	13.07	0.298	0.666	0.727	0.338
DMI, g/d	387.93	386.80	390.28	374.09	18.312	0.924	0.645	0.684
ADG, g/d	41.23	44.94	51.36	45.93	2.637	0.074	0.091	0.092
F/G	9.63^a^	8.85^ab^	7.61^b^	8.30^b^	0.438	0.017	0.011	0.102

^a,^^b^ Different superscripts in the same row indicate significant differences (*P* < 0.05)

^1^CON, basal diet; 200PSE, basal diet + 200 mg/kg DM/d PSE; 400PSE, basal diet + 400 mg/kg DM/d PSE; 600PSE, basal diet + 600 mg/kg DM/d PSE (*n* = 9)

^2^*IBW* Initial body weight, *FBW* Final body weight, *DMI* Dry matter intake, *ADG* Average daily gain, *F/G* Feed to gain ratio = DMI/ADG

Dietary PSE had no effect on slaughter performance of Hainan black goats (*P* > 0.05, Table 2). Meat quality parameters were significantly affected by dietary PSE supplementation. The values of L*_24h_ and b*_24h_ showed significant linear responses to increasing PSE levels, whereas pH_24h_ increased linearly with PSE supplementation (*P* < 0.05, Table 3). Moreover, cooking loss and drip loss decreased linearly with increasing PSE levels (*P* < 0.05). Compared with the CON group, 600 mg/kg PSE supplementation increased pH_24h_ by 4.8%, but decreased drip loss and cooking loss by 5.8% and 23.8%, respectively. No significant differences were observed in moisture, crude ash, crude protein, or ether extract (*P* > 0.05).

**Table 2:** Effect of dietary supplementation with PSE on slaughter performance of Hainan black goats

Items	Groups^1^				SEM	*P*-value		
	CON	200PSE	400PSE	600PSE		Treatment	Linear	Quadratic
Live weight, kg	12.02	12.94	12.91	12.82	0.631	0.698	0.410	0.426
Head weight, kg	1.13	1.22	1.20	1.21	0.106	0.727	0.471	0.472
Hooves weight, kg	0.43	0.45	0.47	0.44	0.022	0.665	0.652	0.265
Fur weight, kg	1.03	1.10	1.14	1.09	0.071	0.742	0.526	0.383
Carcass weight, kg	5.36	5.69	5.72	5.84	0.357	0.799	0.361	0.761
Dressing percentage, %	44.21	43.95	43.85	45.25	1.837	0.762	0,522	0.431
Bone weight, kg	1.76	1.75	1.80	1.86	0.096	0.841	0.408	0.747
Net meat weight, kg	3.60	3.94	3.92	3.98	0.286	0.769	0.387	0.626
Pure meat percentage, %	29.57	30.43	29.91	30.77	1.053	0.855	0.515	0.996
Eye muscle area, cm^2^	7.37	8.11	6.92	7.26	0.893	0.815	0.709	0.826
GR value, mm	4.08	4.07	3.42	3.82	0.290	0.347	0.281	0.491

^1^CON, basal diet; 200PSE, basal diet + 200 mg/kg DM/d PSE; 400PSE, basal diet + 400 mg/kg DM/d PSE; 600PSE, basal diet + 600 mg/kg DM/d PSE (*n* = 9)

**Table 3:** Effect of dietary supplementation with PSE on the meat quality and nutritional composition of Hainan black goats

Items	Groups^1^				SEM	*P*-value		
	CON	200PSE	400PSE	600PSE		Treatment	Linear	Quadratic
Meat quality^2^								
pH_45min_	6.60	6.68	6.53	6.61	0.217	0.766	0.836	0.965
L*_45min_	36.85	37.57	38.30	37.68	0.924	0.743	0.440	0.472
a*_45min_	6.61	5.89	5.89	5.62	0.320	0.943	0.733	0.849
b*_45min_	5.06^b^	7.51^a^	7.25^a^	7.13^a^	0.595	0.023	0.032	0.038
pH_24h_	5.65^b^	5.75^ab^	5.76^ab^	5.92^a^	0.064	0.046	0.008	0.618
L*_24h_	46.58^a^	43.45^b^	42.63^b^	41.28^b^	0.908	0.002	< 0.001	0.332
a*_24h_	4.32	4.56	4.40	3.95	0.450	0.804	0.538	0.444
b*_24h_	10.14	8.70	8.61	8.60	0.507	0.106	0.045	0.168
Cooking loss, %	47.27	47.21	46.15	44.53	0.925	0.146	0.031	0.402
Drip loss, %	2.61	2.46	2.03	1.99	0.188	0.062	0.010	0.760
Nutritional composition^3^								
Moisture, %	77.07	76.12	77.90	76.94	0.688	0.353	0.654	0.992
Ash, %	6.08	6.57	6.77	6.31	0.278	0.331	0.486	0.096
Protein, %	19.12	20.57	20.05	20.79	0.538	0.168	0.073	0.518
Ether extract, %	2.98	3.59	3.15	3.14	0.195	0.196	0.974	0.118

^a,b^ Different superscripts in the same row indicate significant differences (*P* < 0.05)

^1^CON, basal diet; 200PSE, basal diet + 200 mg/kg DM/d PSE; 400PSE, basal diet + 400 mg/kg DM/d PSE; 600PSE, basal diet + 600 mg/kg DM/d PSE

^2^Samples: CON, 200PSE, 400PSE, and 600PSE, *n* = 9

^3^Samples: CON, 200PSE, *n* = 7; 400PSE, 600PSE, *n* = 9

### *Longissimus lumborum* muscle metabolic reprogramming induced by PSE

**Fig. 1:**
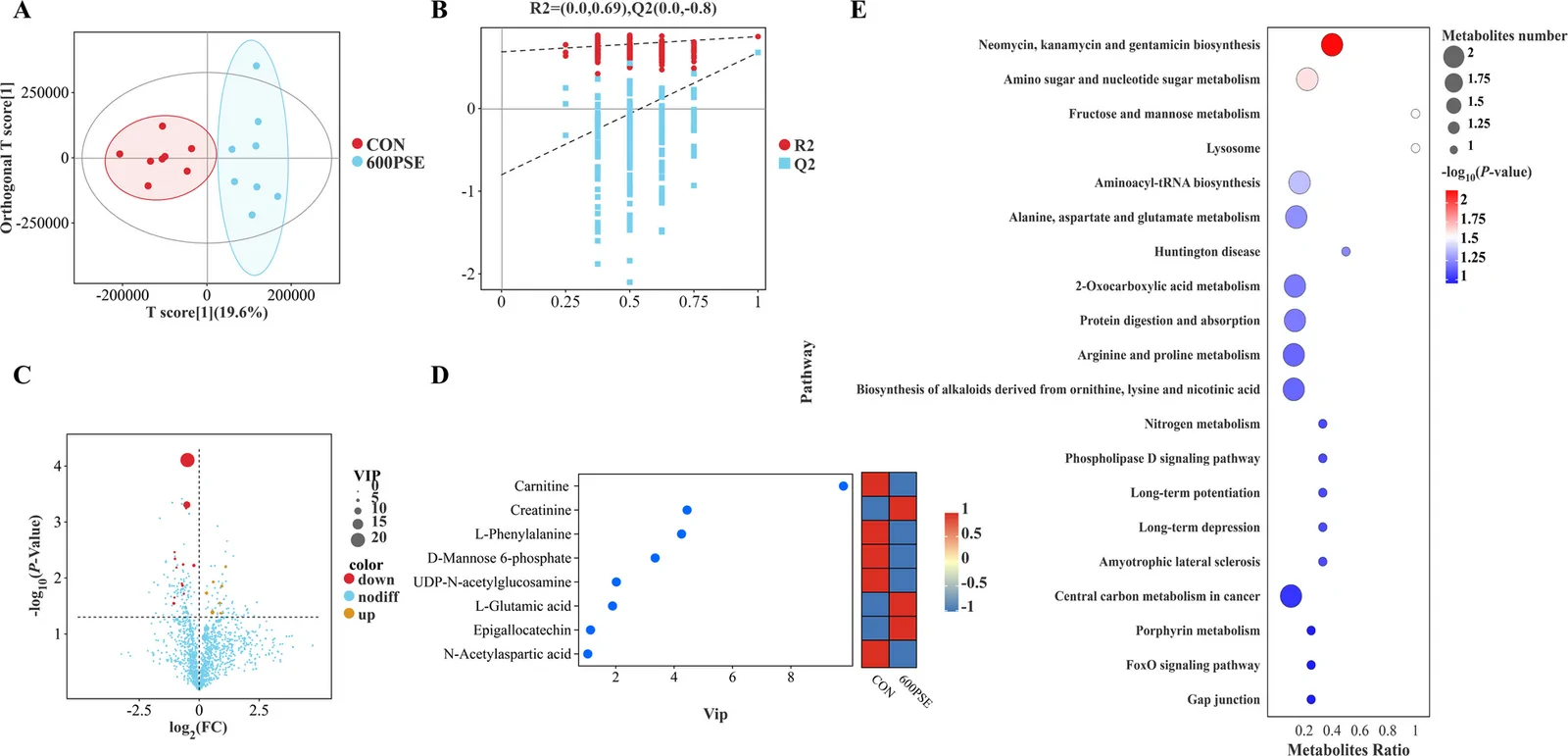
Differential muscle metabolites between CON and 600PSE group. **A** OPLS-DA score plots are based on metabolomics data. **B** The permutation test of OPLS-DA between CON and 600PSE group. **C** The Volcano plot based on DMs. **D** The Vip of DMs between CON and 600PSE group. **E** Differential KEGG enriched from DMs

Based on the linear effect differences in meat quality traits, the CON and 600 mg/kg PSE groups were selected for subsequent mechanistic analyses. A total of 1,436 muscle metabolites were detected, primarily classified as organic acids and derivatives, lipids, and organoheterocyclic compounds (Fig. S2). OPLS-DA analysis revealed a clear separation between the CON and 600PSE groups (Fig. 1A), which was validated by permutation testing (Q^2^ = 0.68; Fig. 1B). A total of 21 differential metabolites (DMs) were identified, including 8 upregulated and 13 downregulated metabolites (Fig. 1C, Table S3). KEGG enrichment analysis indicated that the DMs were mainly involved in amino acid metabolism and carbohydrate metabolism pathways (Fig. S2), including amino sugar and nucleotide sugar metabolism (*P* = 0.023), fructose and mannose metabolism (*P* = 0.028), alanine, aspartate and glutamate metabolism (*P* = 0.053), and 2-oxocarboxylic acid metabolism (*P* = 0.061, Fig. 1E). Notably, metabolites (Fig. 1D) associated with these pathways were significantly altered. In particular, L-glutamic acid and creatinine were increased, whereas N-acetyl-L-aspartic acid, UDP-N-acetylglucosamine, and D-mannose 6-phosphate were decreased, suggesting a coordinated alterations in amino acid and energy-related metabolic pathways in muscle tissue. The elevation of glutamic acid was further validated by targeted metabolomics, confirming its consistent upregulation (Fig. S2).

**Fig. 2:**
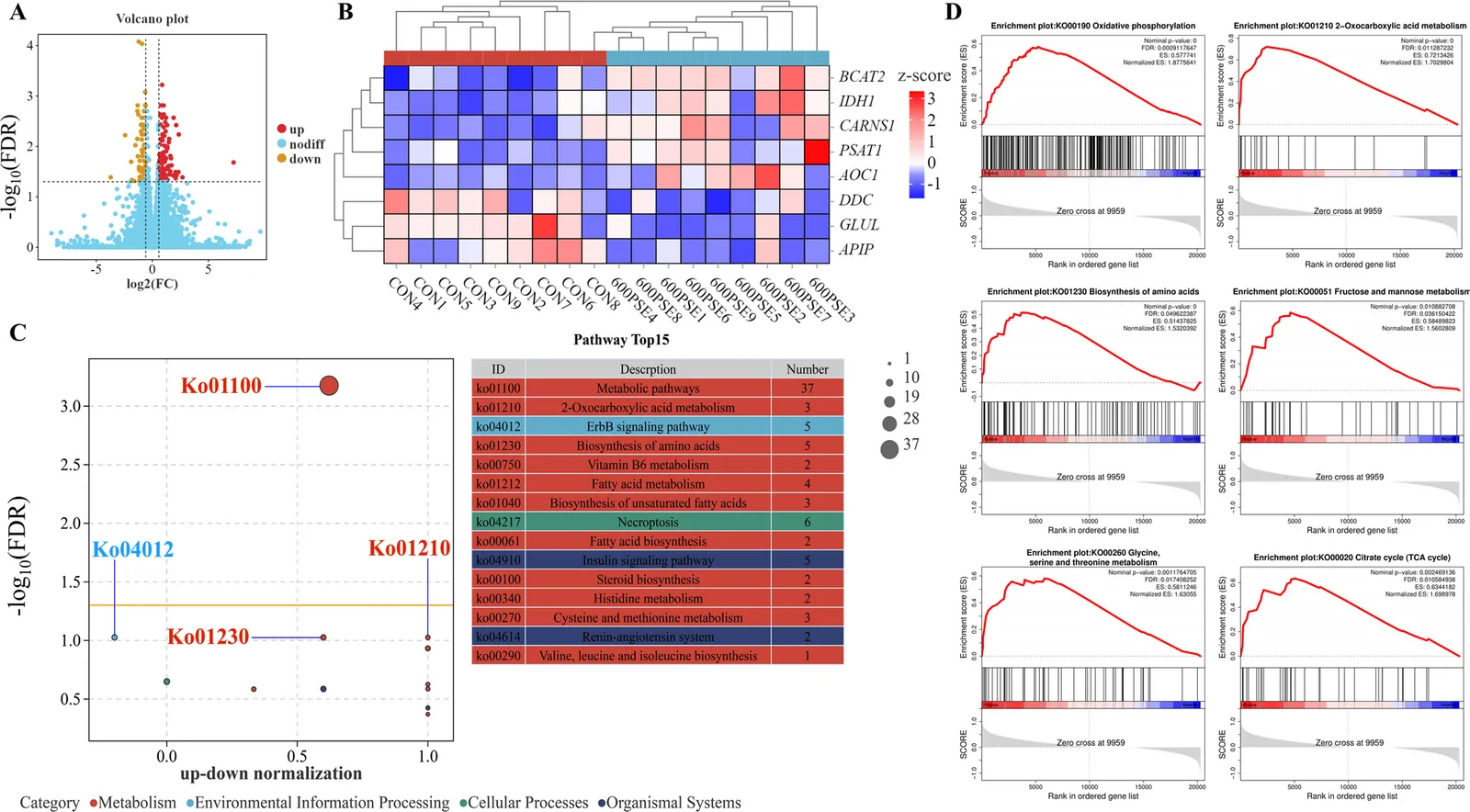
Differential transcriptomics between CON and 600PSE groups. **A** The volcano plot based on DEGs. **B** The heatmap of DEGs associated with amino acids metabolism between CON and 600PSE groups. **C** Differential KEGG enriched from DEGs. **D** GSEA highlighting significantly enriched pathways (FDR < 0.05, *|*NES*| *> 1)

A total of 196 DEGs were identified between the CON and 600PSE groups (FDR < 0.05, |log_2_FC| > 0.58, Table S4), including 124 upregulated and 72 downregulated genes (Fig. 2A). The qPCR validation confirmed the reliability of RNA-seq data, showing consistent expression trends for selected genes (Fig. S1). Several genes related to amino acid metabolism were significantly altered, including upregulation of *PSAT1*, *AOC1*, *CARNS1*, *BCAT2*, and *IDH1*, and downregulation of *APIP*, *DDC*, and *GLUL* (Fig. 2B). Further KEGG analysis demonstrated that DEGs were predominantly enriched in metabolic pathways (Fig. 2C), particularly 2-oxocarboxylic acid metabolism (FDR = 0.094), ErbB signaling pathway (FDR = 0.094), and biosynthesis of amino acids (FDR = 0.094). GSEA further revealed a global activation of metabolic pathways in the 600PSE group, with 279 of 332 enriched pathways being upregulated. Among these, key pathways involved in amino acid metabolism and energy metabolism, including oxidative phosphorylation, 2-oxocarboxylic acid metabolism, biosynthesis of amino acids, fructose and mannose metabolism, glycine, serine and threonine metabolism, and citrate cycle (TCA cycle) were significantly upregulated in the 600PSE group (FDR < 0.05, |NES| > 1, Fig. 2D).

### Differences in rumen metabolome induced by PSE

**Fig. 3:**
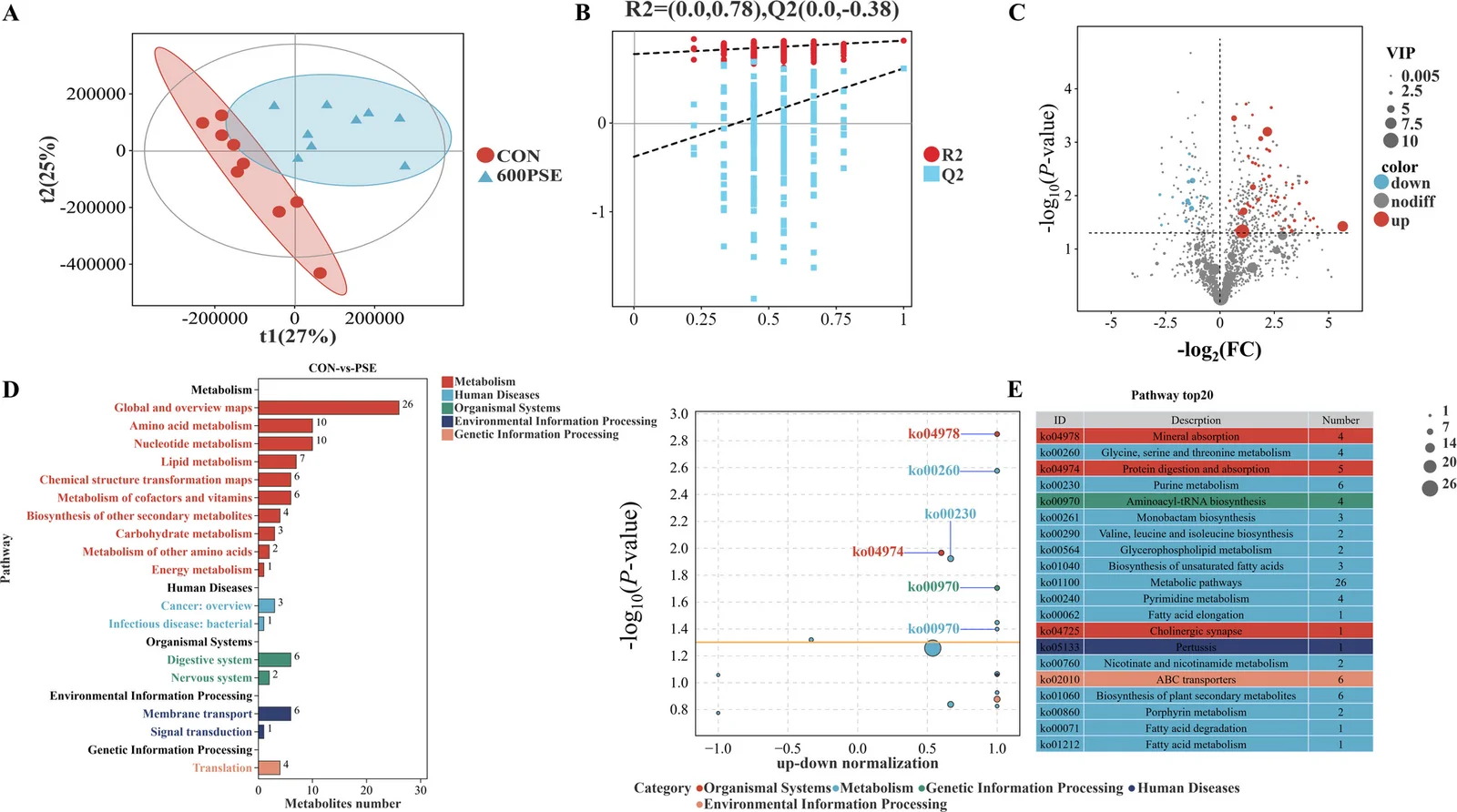
Differential ruminal metabolites between CON and 600PSE groups. **A** PLS-DA score plots are based on metabolomics data. **B** The permutation test of PLS-DA. **C** The volcano plot based on DMs. **D** KEGG enrichment analysis of DMs. **E** Differential KEGG enriched from DMs

A total of 1,466 rumen metabolites were detected in the mixed ion mode (Positive + Negative) analysis. PLS-DA revealed a clear separation between the CON and 600PSE groups, which was validated by permutation testing (Q^2^ = 0.62; Fig. 3A and B). A total of 68 differential metabolites were identified, including 52 upregulated and 16 downregulated metabolites (Fig. 3C). KEGG enrichment analysis indicated that these DMs were primarily involved in digestive system, amino acid and nucleotide metabolism (Fig. 3D). Specifically, glycine, serine and threonine metabolism and valine, leucine and isoleucine biosynthesis, mineral absorption, protein digestion and absorption, and purine metabolism were significantly enriched (*P* < 0.05 Fig. 3E). Consistently, key metabolites associated with these pathways were increased (Table S5), including L-threonine, L-serine, L-methionine, and choline, along with purine derivatives, whereas indole was significantly decreased. These alterations suggest that PSE supplementation promotes amino acid metabolic activity in the rumen and enhances the production of amino acid precursors.

### Differences in rumen microbiota induced by PSE

**Fig. 4:**
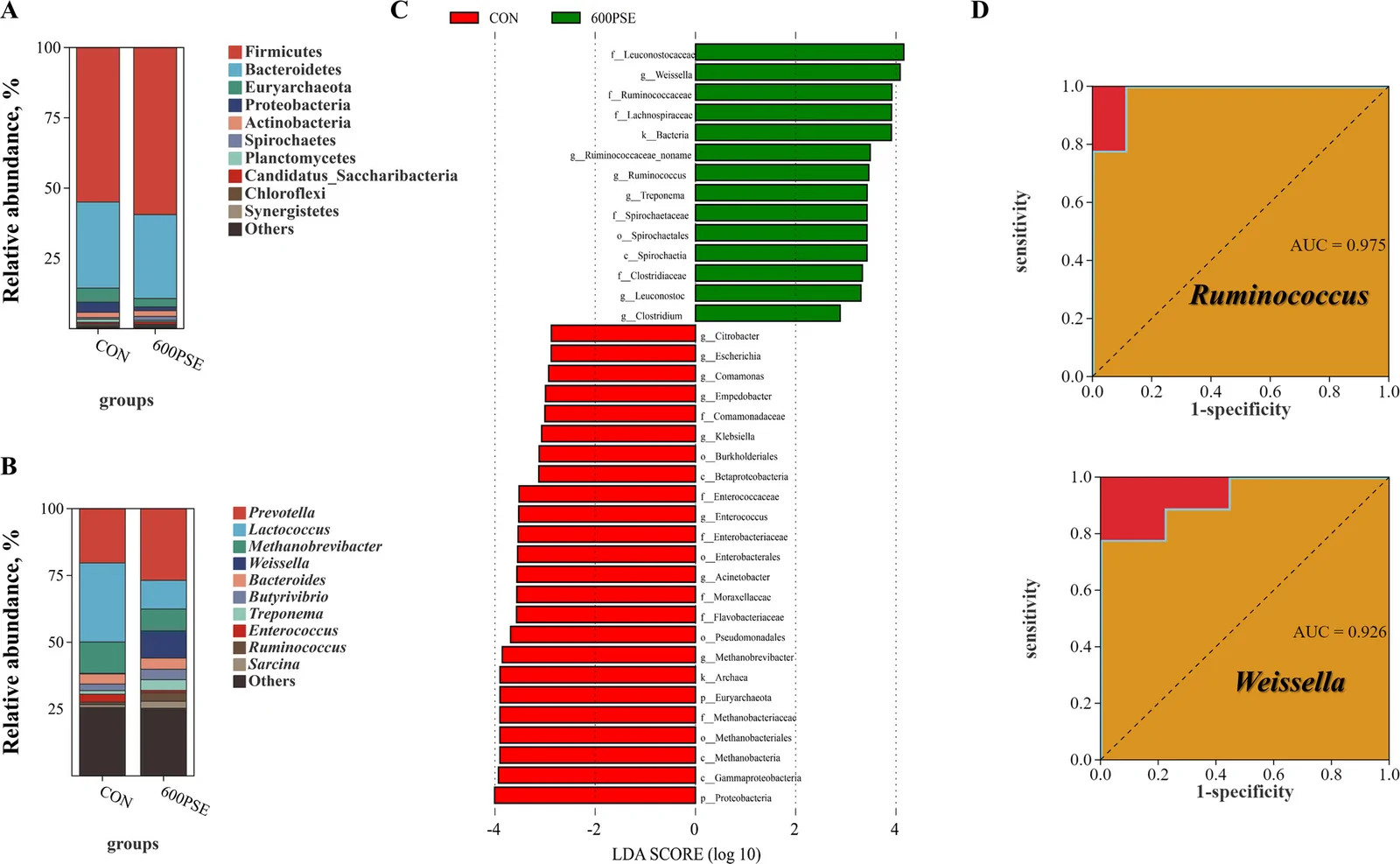
Differential microbial analysis on rumen metagenomics of Hainan black goats induced by PSE. **A** Rumen metagenomic microbial phylum-level analysis. **B** Rumen metagenomic microbial genus-level analysis. **C** LEfSe analysis of rumen metagenomic species differences in Hainan black goats induced by PSE (LDA > 2). **D** ROC curve analysis of differential microbial taxa identified from rumen metagenomic sequencing. The area under the curve (AUC > 0.9) was calculated to evaluate the discriminatory performance of individual taxa between the CON and 600PSE groups

Based on metagenomic sequencing, no significant differences in α diversity indices (Sob, Shannon, and Simpson) were observed between the CON and PSE600 groups (*P* > 0.05; Fig. S3). In contrast, β diversity analysis based on Bray–Curtis distances at the family-level revealed a significant alteration in microbial community structure (ANOSIM, R^2^ = 0.157, *P* = 0.013), which was further supported by distinct clustering in PCoA analysis ( Fig. S3). These results suggest that dietary PSE modifies the taxonomic composition of the rumen microbiome without affecting overall diversity, potentially leading to shifts in microbial metabolic functions. At the phylum level of the rumen metagenome (Fig. 4A and B), Firmicutes and Bacteroidetes were the dominant phyla. In addition, the dominant genera were primarily *Prevotella* and *Lactococcus*. Compare to the CON group, further LEfSe analysis (Fig. 4C, Table S6) revealed that the relative abundances of *Acinetobacter*, *Enterococcus*, *Klebsiella*, and *Methanobrevibacter* (LDA > 3) were significantly lower than those in the 600PSE group; conversely, the relative abundances of *Leuconostoc*, *Ruminococcaceae_noname*, *Ruminococcus*, *Treponema*, and *Weissella* (LDA > 3) were significantly higher in the 600PSE group. Moreover, the AUC values of *Ruminococcus* and *Weissella* were 0.975 and 0.926 (Fig. 4D), respectively, indicating strong discriminatory performance between the CON and 600PSE groups. These findings suggest that the altered abundances of *Ruminococcus* and *Weissella* may serve as potential microbial indicators associated with PSE supplementation-induced changes in the rumen microbiota.

**Fig. 5:**
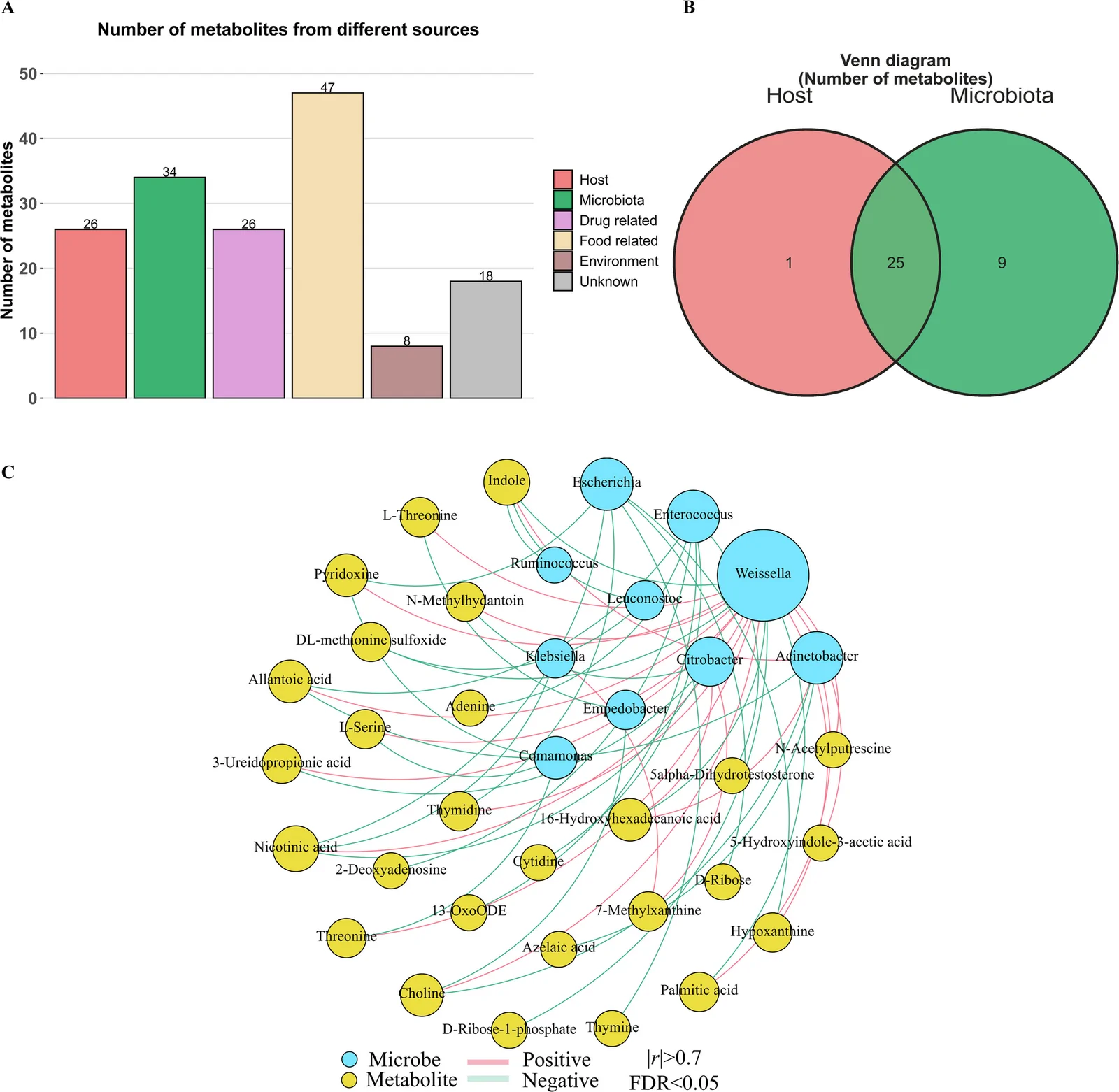
Potential origins of differential rumen metabolites and microbial–metabolite correlation networks in Hainan black goats induced by PSE. **A** Histogram of potential origins of differential rumen metabolites. **B** Venn diagram showing the overlap of potential metabolite origins. **C** Correlation network analysis of key differential metagenomic microbes (genus level) and differential ruminal metabolites of the rumen in Hainan black goats (FDR < 0.05, |*r*| > 0.7)

Among differential rumen metabolites, potential origins were annotated into six categories: host, microbiota, food, drug, environmental, and unknown sources using MetOrigin 2.0 (Fig. 5 A and B). Notably, several key metabolites, including choline, L-threonine, and L-serine, showed overlapping potential origins, indicating that their occurrence in the rumen may reflect complex interactions among host metabolism, microbial metabolism, and dietary intervention (Table S7). Further correlation network analysis revealed that *Weissella* occupies a central position in the key “microbe-metabolite” interaction network and is significantly correlated with multiple DMs (Fig. 5C). Specifically, *Weissella* showed a significant positive correlation with the upregulated L-serine, L-threonine, choline, hypoxanthine, L-methionine, and 2-hydroxyvaleric acid in the rumen metabolites; among these, serine, threonine, and choline were significantly enriched in the glycine, serine, and threonine metabolism pathway within the KEGG metabolic network; while showing a significant negative correlation with the downregulated rumen metabolites D-ribose-1-phosphate, methionine sulfoxide, palmitic acid, docosapentaenoic acid, and indole; *Ruminococcus* and *Leuconostoc*, which were significantly enriched in the 600PSE group, showed a significant negative correlation with indole. These findings indicate that PSE supplementation reshapes rumen microbial ecology and promotes amino acid-associated metabolic interactions, with *Weissella* as a potential key mediator.

**Fig. 6:**
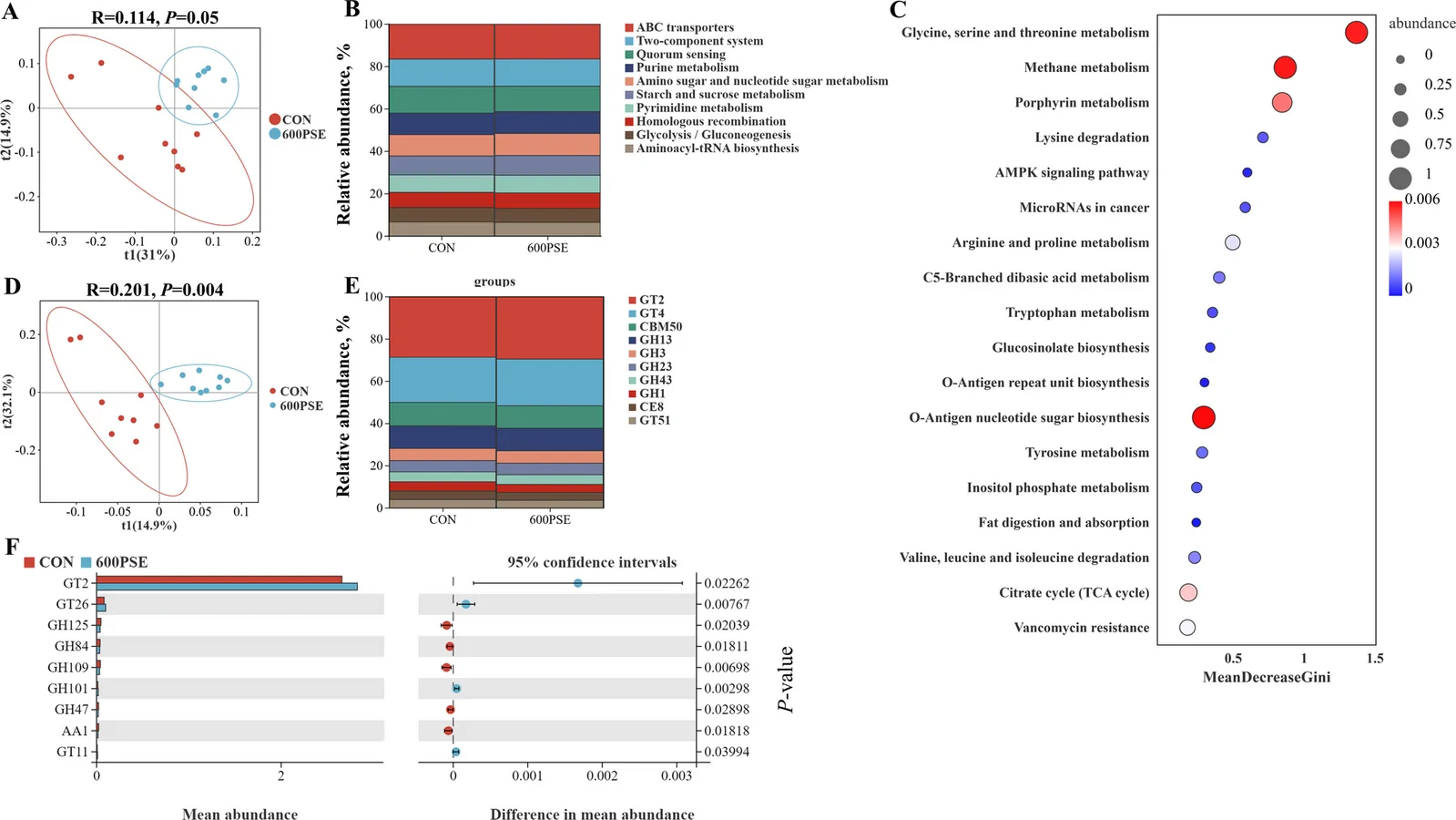
Differential functional annotation and analysis of the rumen metagenome of Hainan black goats between CON and 600PSE groups. **A** PCoA of differences in KEGG functional pathway-level β diversity. **B** Top ten KEGG pathway by relative abundance. **C** PCoA of differences in CAZy functional level-B β diversity. **D** Top ten CAZy level-B by relative abundance. **E** Differences in CAZymes level-B. **F** Rumen metagenomic random forest biomarker KEGG pathway analysis

Based on KEGG functional annotation of metagenomic genes, PLS-DA analysis revealed no significant differences in β diversity at the pathway level (ANOSIM, R^2^ = 0.114, *P* = 0.05, Fig. 6A). The dominant KEGG pathways were ABC transporters and Two-component system (Fig. 6B). Random forest model revealed that glycine, serine, and threonine metabolism was the most differential metabolic pathway distinguishing the CON group from the 600PSE group (Fig. 6C), suggesting that dietary PSE may influence the metabolic potential of rumen microbes by regulating amino acid-related metabolic functions. Dietary PSE significantly altered the β diversity of ruminal CAZymes at the level B (ANOSIM, R^2^ = 0.114, *P* = 0.05, Fig. 6D). Based on CAZymes functional annotation analysis and Welch’s *t*-test (Fig. 6E and F), the relative abundances of GT2, GT26, GH101, and GT11 in the CAZy family were significantly higher in the 600PSE group than in the CON group; conversely, the relative abundances of AA1, GH119, GH110, GH93, and GT18 were significantly higher in the CON group than in the 600PSE group.

### Identification of key functional microbe through metagenomic binning

**Fig. 7:**
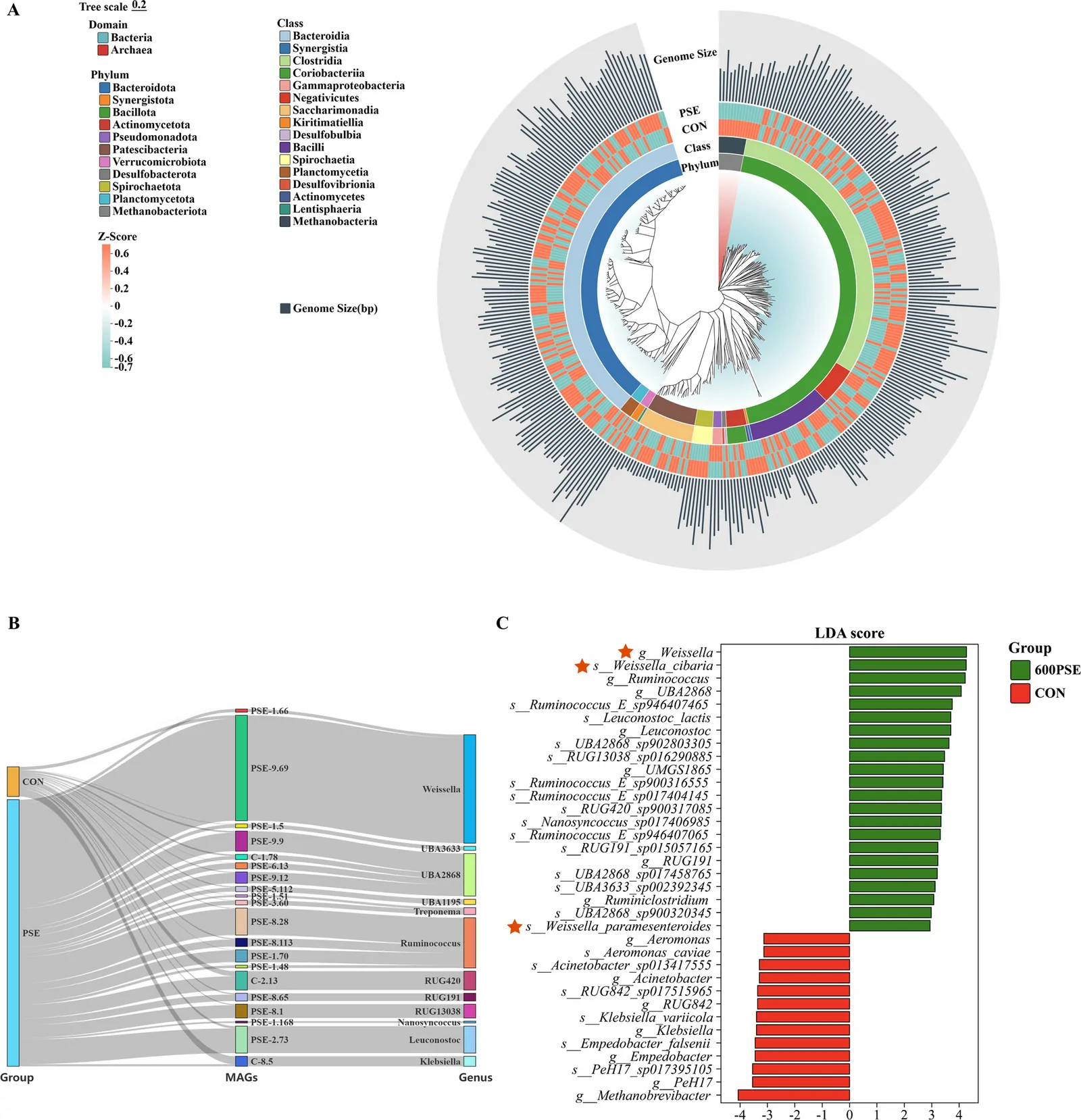
Phylogenetic and differential abundance analyses of the rumen microbiome based on high-quality MAGs. **A** Phylogenetic tree of high-quality MAGs sampled from the rumen in Hainan black goats. Branch nodes are colored according to the microbial domain level corresponding to MAGs. Inner concentric layers are colored according to the phylum and class levels to which MAGs belong. The middle concentric layer heatmap represents microbial relative abundance (calibrated using Z-scores). The outer concentric layers denote microbial genome size (bp). **B** Species differences Sankey diagram from rumen high-quality MAGs. **C** LEfSe analysis from rumen high-quality MAGs (LDA > 3)

A phylogenetic tree was constructed based on recovered 361 high-quality MAGs (Fig. 7A), and including 350 bacterial and 11 archaeal genomes. At the Bacteria phylum level, the rumen microbiome was dominated by Bacillota (Firmicutes) and Bacteroidota (Bacteroidetes‌). Consistent with metagenomics, differential analysis at the species-level further identified several functionally relevant Mags. Notably, *Weissella* and *Ruminococcus* were significantly enriched in the 600PSE group, whereas *Klebsiella* was more abundant in the CON group (Fig. 7B). LEfSe analysis confirmed that *Weissella cibaria* (PSE-9.69, completeness = 97.29%, contaminatio*n* = 3.51%) and *Weissella paramesenteroides* (PSE-1.66, completeness = 81.53%, contaminatio*n* = 4.13%) were significantly enriched in the 600PSE group (Fig. 7C).

**Fig. 8:**
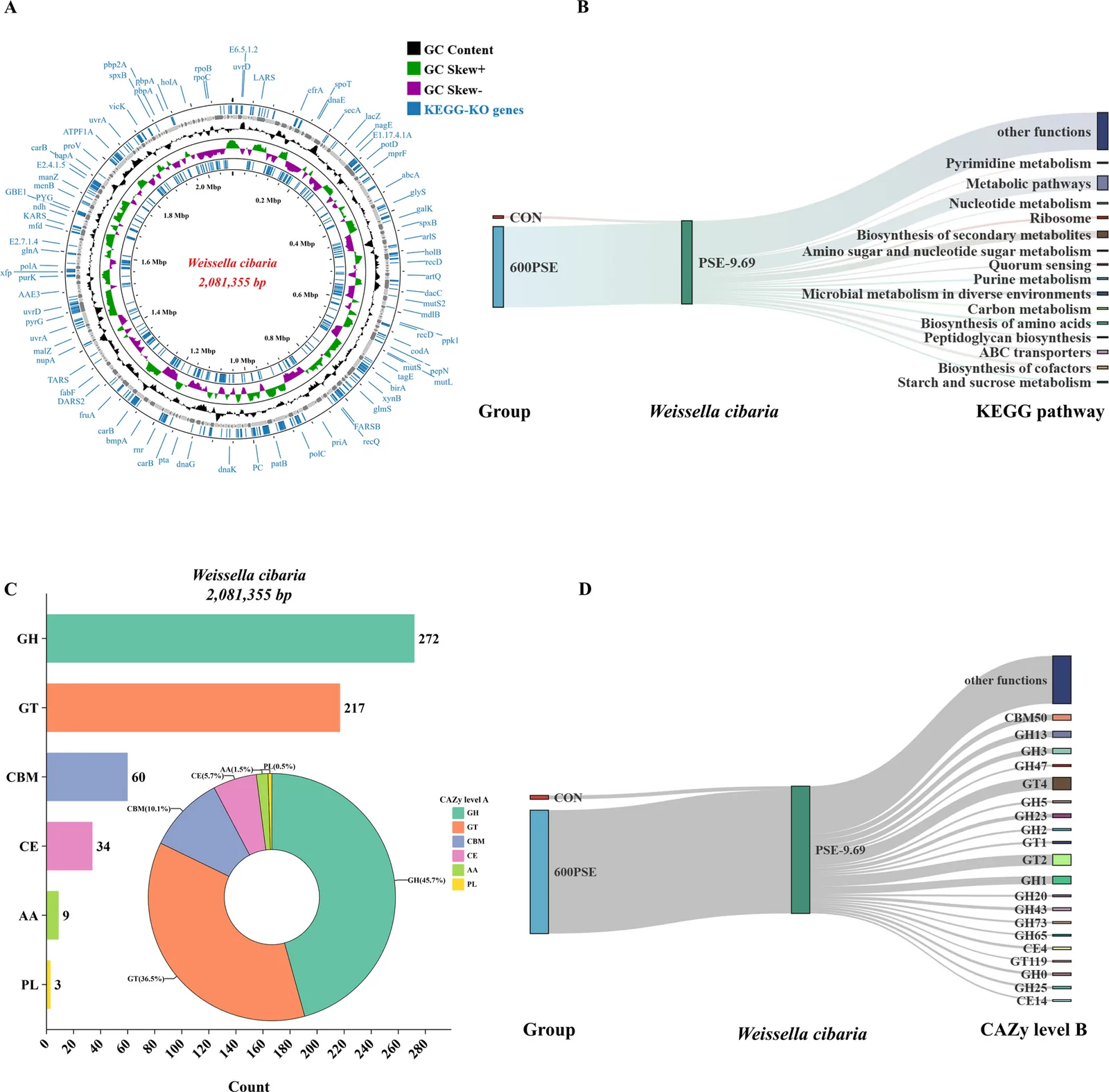
High-quality MAGs single-bacterial gene cluster diagrams and functional gene metabolic pathway annotations based on KEGG and CAZy database. **A** The gene cluster diagrams of *Weissella cibaria*. **B** Top 15 KEGG pathway annotations enriched by functional gene in *Weissella cibaria*. **C** The CAZymes activity cluster diagrams of *Weissella cibaria*. **D** Top 20 CAZymes families activity enriched by functional gene in *Weissella cibaria*

To further explore the functional potential of key microbes, *Weissella cibaria* (PSE-9.69) was selected for in-depth analysis of its genomic structural characteristics and potential metabolic functions. Based on gene prediction and functional annotation results, the MAG of *Weissella cibaria* was visualized using the Proksee platform (https://proksee.ca/, Fig. 8A). KEGG functional annotation results indicate that a total of 664 functional genes were annotated in this MAG. It showed that *Weissella cibaria* primarily enriched in metabolic pathways, biosynthesis of secondary metabolites, and biosynthesis of amino acids (Fig. 8B).Among them, seven key functional genes were annotated in the glycine, serine, and threonine metabolism pathway, namely *hom* (K00003), *thrB* (K00872), *glxK* (K00865), *lysC* (K00928), *PGAM* (K01834), *glyA* (K00600), and *asd* (K00133), indicating that this strain possesses a relatively complete set of genes encoding these amino acid metabolic pathways. Notably, *lysC* (K00928) and *glyA* (K00600) were significantly upregulated. Integration with metabolomic data further revealed consistent alterations in related metabolites within these pathways (Fig. S4), including choline, creatine, L-threonine, guanidineacetic acid, O-phospho-L-serine, L-aspartic acid, and L-serine. Specifically, choline, L-threonine, and L-serine were significantly upregulated in the 600PSE group, consistent with the functional gene annotation results for this strain in the relevant amino acid metabolic pathways. These results suggest that *Weissella cibaria* may contribute to PSE-induced enhancement of amino acid metabolism in the rumen.

In addition, a total of 595 CAZy-related genes were identified in *Weissella cibaria* (Fig. 8C), with glycoside hydrolases (GH, 45.7%) and glycosyltransferases (GT, 36.5%) representing the dominant classes. Sankey plot further showed that *Weissella cibaria* contributed substantially to CAZy functional profiles in the 600PSE group (PSE = 9.69), mainly associated with increased abundances of multiple CAZy GT families (Fig. 8D).

### Associations between rumen microbiota and metabolites, muscle metabolites and genes, and meat quality index

**Fig. 9:**
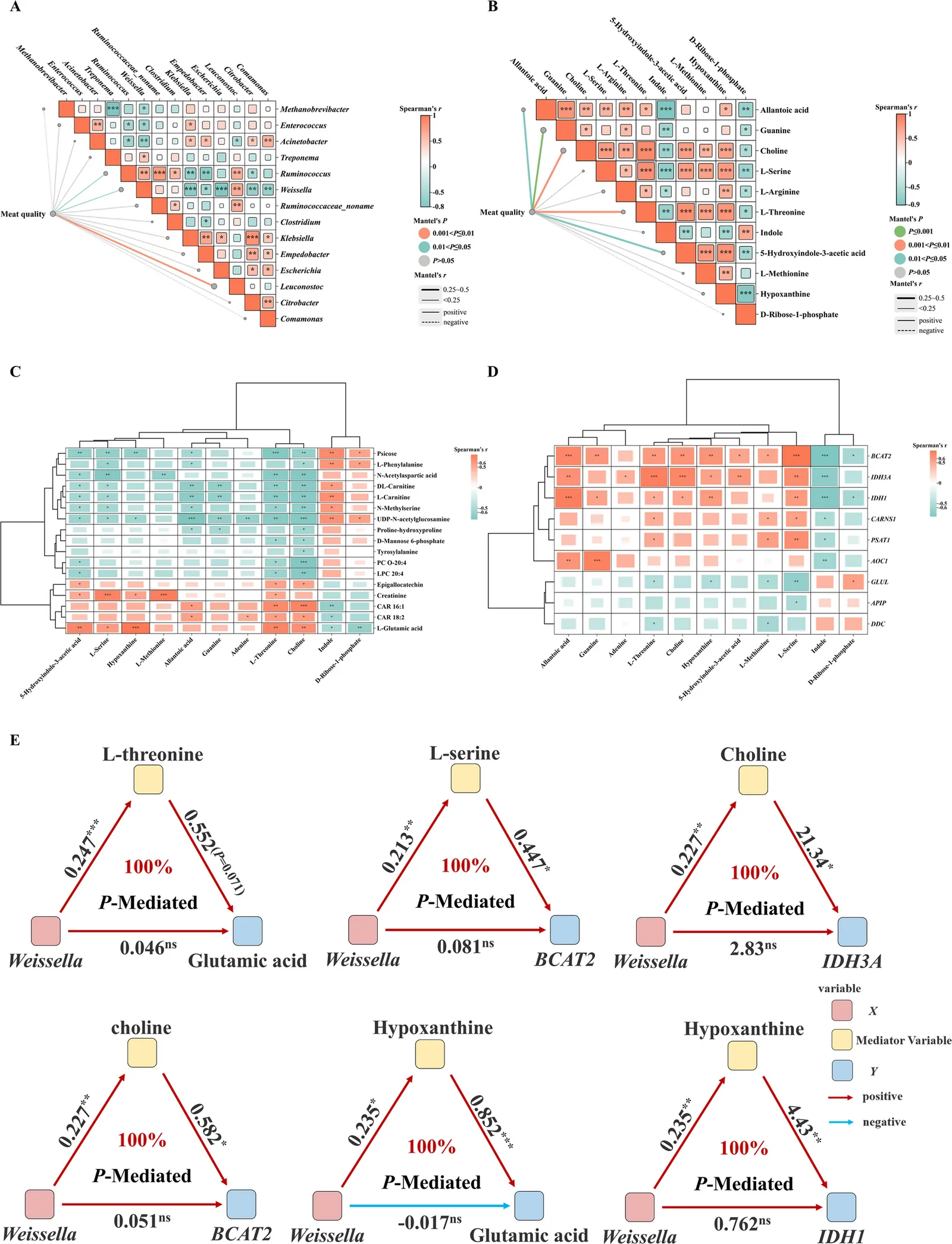
Integrated association linking rumen microbiota, rumen metabolites, muscle metabolites, muscle transcriptome genes, and host meat quality traits. **A** Mantel test of the key ruminal microbes and meat quality. **B** Mantel test of the key DMs and meat quality. **C** Spearman's correlation analysis of key DMs in the rumen and muscle. **D** Spearman's correlation analysis of key ruminal DMs and muscle transcriptome genes. **E** Mediation analysis of key rumen microbes and metabolites, and differentially expressed metabolites/genes in the *longissimus lumborum * muscle of Hainan black goats in the CON and 600PSE groups. ^*^P < 0.05, ^**^P < 0.01, ^***^P < 0.001; ns, not significant

Mantel tests were conducted to assess the overall relationships between rumen microbial/metabolic profiles and meat quality traits. The Mantel test revealed a significant association between rumen microbial/metabolite composition and meat quality parameters, indicating a potential microbiota–host metabolic linkage (Fig. 9A and B). At the genus level, *Ruminococcus* (*r* = 0.205, *P* = 0.042), *Weissella* (*r* = 0.233, *P* = 0.039), and *Leuconostoc* (*r* = 0.320, *P* = 0.009) were significantly associated with variations in meat quality (*P* < 0.05), suggesting their involvement in host metabolic regulation. At the metabolite level, several rumen-derived metabolites, including choline, L-threonine, L-serine, guanine, and allantoic acid, were significantly correlated with meat quality traits. These metabolites are primarily involved in amino acid and nucleotide metabolism, indicating that rumen metabolic activity may contribute to the modulation of meat quality.

To further elucidate the potential metabolic crosstalk between the rumen and muscle, correlation analysis was performed between key rumen metabolites and muscle metabolic and transcriptomic profiles (Fig. 9C and D). Notably, several rumen metabolites, including L-serine, L-threonine, choline, and 5-hydroxyindole-3-acetic acid, exhibited strong positive correlations with muscle L-glutamic acid, whereas D-mannose 6-phosphate and indole showed negative correlations. This suggests that rumen-derived metabolites may contribute to the accumulation of glutamic acid in muscle. Furthermore, these metabolites were also significantly associated with key genes involved in amino acid and 2-oxocarboxylic acid metabolism. In particular, L-serine, L-threonine, and choline showed strong positive correlations with *BCAT2* and *IDH3A*, while indole exhibited strong negative correlations. Hypoxanthine showed strong positive significant correlations with genes associated with *BCAT2* and *IDH1*. These genes are critical regulators of amino acid transamination and energy metabolism, indicating a coordinated regulation of metabolic flux. Collectively, these findings support a potential mechanistic link in which rumen-derived metabolites modulate muscle glutamate metabolism and its associated gene expression, thereby contributing to PSE-induced metabolic reprogramming and meat quality improvement.

Based on the correlation analysis, mediation models were constructed to evaluate the potential relationships among microbes, metabolites, and host muscle metabolism (Fig. 9E). Six significant mediation pathways were identified, all centered on the key differential genus *Weissella*, including: *Weissella* → L-threonine → glutamic acid, *Weissella* → hypoxanthine → glutamic acid, *Weissella* → L-serine → *BCAT2*, *Weissella* → choline → *BCAT2*, *Weissella* → choline → *IDH3A*, and *Weissella* → hypoxanthine → *IDH1*. Notably, pathways involving L-threonine and hypoxanthine mediated the association between *Weissella* and muscle glutamic acid, indicating their role in regulating glutamate metabolism. In parallel, L-serine, choline, and hypoxanthine mediated the association between *Weissella* and key genes involved in glutamate metabolism, including *BCAT2*, *IDH3A*, and *IDH1*, suggesting coordinated regulation at the transcriptional level. In all models, the indirect effects were significant, whereas the direct effects were not, indicating statistical mediation. These findings support a potential mechanism in which *Weissella* may influence muscle glutamate metabolism through metabolite-mediated pathways linking rumen microbial activity to host metabolic and transcriptional responses. This highlights a metabolite-mediated microbiota–muscle axis centered on glutamate metabolism.

## Discussion

**Fig. 10:**
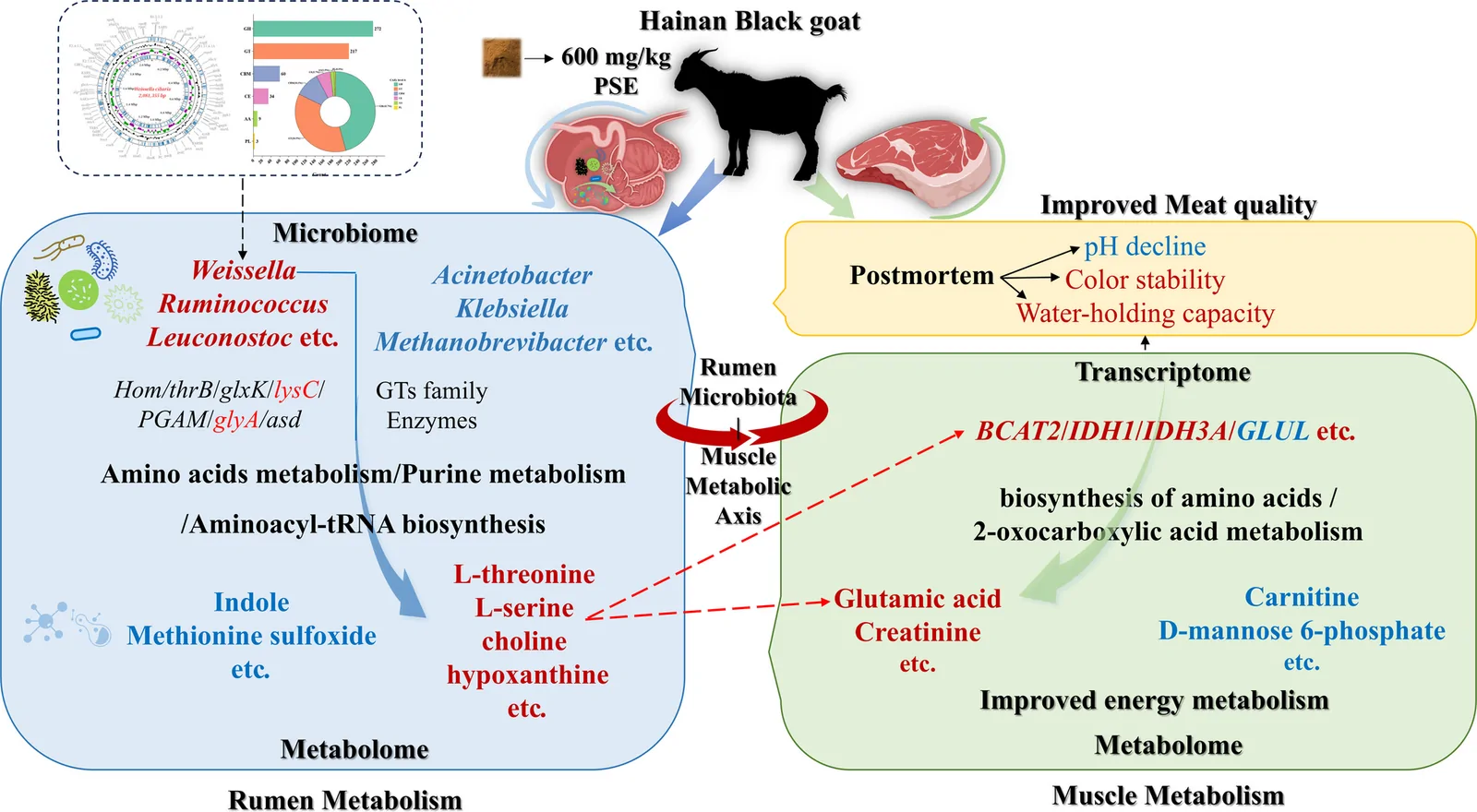
Proposed association network between PSE-induced rumen microbial alterations, microbial metabolites, and host muscle metabolic responses. PSE supplementation was associated with changes in rumen microbial composition, including an increased abundance of key functional taxa such as *Weissella*, together with alterations in microbial CAZy activity. These microbial functional changes were accompanied by variations in amino acid biosynthesis and nitrogen metabolism-related pathways, as well as altered levels of rumen metabolites, including serine, threonine, choline, and hypoxanthine. Correlation and mediation analyses suggested that these microbial taxa and metabolites were statistically associated with muscle metabolic remodeling, particularly glutamate metabolism-related changes. These findings support a potential microbiota–metabolite–host metabolic association network that may contribute to postmortem energy metabolism and meat quality traits, including higher ultimate pH, improved water-holding capacity, and enhanced color stability

The present study provides integrated multi-omics evidence indicating that dietary PSE supplementation is associated with coordinated changes in the rumen microbiota, microbial metabolites, and host muscle metabolic profiles in relation to meat quality improvement. Although growth performance and carcass traits were not markedly affected, improvements in meat quality parameters, including a higher pH at 24 h postmortem, enhanced color stability, and improved water-holding capacity, were observed. Furthermore, genome-resolved metagenomic analysis identified a *Weissella cibaria* MAG containing genes associated with amino acid metabolism and carbohydrate-active enzyme functions, suggesting its potential involvement in rumen metabolic alterations; Collectively, these findings support a potential microbiota–metabolite–muscle metabolic association network associated with PSE-induced improvements in goat meat quality (Fig. 10).

Postmortem energy metabolism is a critical determinant of meat physicochemical properties, as the rate of ATP depletion governs pH decline and ultimately influences meat color and water-holding capacity [46–48]. Following slaughter, oxygen deprivation induces a shift toward anaerobic glycolysis, leading to lactate accumulation, rapid ATP exhaustion, and a decline in pH [49]. The rate of pH decline critically affects protein denaturation and myoglobin stability, thereby shaping meat quality attributes [50]. In the present study, dietary PSE supplementation was associated with a moderate improvement in postmortem meat quality traits, particularly those related to pH stability and water holding capacity. The improved ultimate pH and reduced moisture loss suggest that PSE may contribute to maintaining muscle physicochemical stability during postmortem conversion. These improvements may have potential implications for reducing purge loss and improving retail quality of goat meat. However, sensory evaluation and shelf-life studies are still required to determine whether these statistical improvements translate into enhanced consumer acceptance. Furthermore, improvements in meat quality traits were closely associated with enhanced amino acid and energy metabolism in muscle, particularly the accumulation of glutamic acid. As a key umami-related amino acid, glutamic acid contributes directly to flavor formation [51, 52] and serves as a central metabolic node linking amino acid metabolism with the tricarboxylic acid (TCA) cycle through transamination reactions [53, 54]. Therefore, increased glutamate availability may contribute to the regulation of postmortem energy metabolism and associated quality traits. Notably, the elevation of glutamic acid was consistently confirmed by both untargeted and targeted metabolomics, indicating a robust enhancement of glutamic acid deposition under PSE supplementation.

In addition, coordinated changes in energy-related metabolites further support a shift in muscle metabolic status. Creatinine, a downstream metabolite of the creatine–phosphocreatine system, reflects enhanced energy buffering capacity and intracellular ATP stabilization [55]. In contrast, carnitine, which is essential for mitochondrial transport of long-chain fatty acids for β-oxidation [56, 57], was decreased. The concomitant increase in creatinine and decrease in carnitine, together with the enrichment of energy metabolism–related pathways identified by GSEA, suggest a metabolic reprogramming characterized by altered energy utilization patterns and enhanced phosphagen buffering capacity in the 600PSE group. Such changes may influence postmortem energy metabolism and contribute to improved meat quality development.

At the transcriptional level, integrated metabolomic and transcriptomic analyses revealed coordinated activation of amino acid and energy metabolism pathways. The upregulation of *BCAT2*, *IDH1*, and *IDH3A*, together with the downregulation of *GLUL*, indicates enhanced amino acid transamination and reduced glutamate reutilization. *BCAT2* catalyzes the reversible transamination of branched-chain amino acids, contributing to glutamate production [58], whereas *IDH1* and *IDH3A* link amino acid metabolism to the TCA cycle through the generation of α-ketoglutarate [59]. These transcriptional changes provide mechanistic support for the observed increase in glutamate levels and indicate enhanced metabolic flux from amino acids into central carbon metabolism. Consistently, enrichment of pathways such as 2-oxocarboxylic acid metabolism and biosynthesis of amino acids further supports the activation of integrated amino acid–energy metabolism in muscle.

Increasing evidence suggests that changes in rumen metabolic activity can be transmitted to peripheral tissues through metabolite-mediated pathways, forming a “rumen–tissue” axis that ultimately influences muscle metabolic processes and product traits [9, 60]. Rumen metabolomics revealed significant enrichment of amino acid-related pathways, particularly glycine, serine, and threonine metabolism, along with increased levels of key metabolites such as serine, threonine, and choline. These metabolites serve as important precursors and intermediates in amino acid biosynthesis and one-carbon metabolism [61, 62]. Their elevated abundance indicates that PSE enhances microbial amino acid metabolism, thereby increasing the availability of metabolically active substrates for host utilization. In contrast, the decreased levels of methionine sulfoxide and indole—a microbial-derived metabolite associated with proteolytic activity and potential toxicity [63]—suggest a shift toward a more favorable rumen metabolic environment with reduced oxidative stress and protein degradation [64, 65]. These results indicate that rumen metabolism acts as an upstream source of key metabolites that support host muscle metabolic remodeling.

Upstream of rumen metabolism, rumen microbial composition was significantly reshaped by PSE supplementation. Although α-diversity remained unchanged, β-diversity analysis demonstrated a clear restructuring of the microbial community. Notably, *Weissella*, *Ruminococcus*, and related taxa were significantly enriched in the 600PSE group, whereas several potentially opportunistic genera, including *Acinetobacter* and *Klebsiella*, were reduced. This pattern is consistent with the reported antimicrobial properties of PSE [66–69]. *Ruminococcus* and *Ruminococcaceae_noname* are well-known cellulolytic and short-chain fatty acid-producing bacteria [70–72], which are typically associated with improved rumen fermentation efficiency and energy supply. In contrast, *Weissella*, a member of the Lactobacillaceae family, has recently gained attention for its roles in carbohydrate utilization and amino acid metabolism, as well as its probiotic potential [73]. In the present study, *Weissella* occupied a central position in the microbe–metabolite interaction network and showed strong positive correlations with amino acid-related metabolites, suggesting a key role in regulating rumen metabolism.

Metagenomic binning analysis further provided functional insights into the role of Weissella. The reconstructed genome of *Weissella cibaria* harbored a relatively complete set of genes involved in glycine, serine, and threonine metabolism, including *glyA* and *lysC*, which are critical for amino acid biosynthesis. The consistency between genomic potential and metabolomic alterations strongly supports the contribution of this strain to enhanced amino acid precursor production in the rumen. Previous studies have also demonstrated that *Weissella* species are involved in amino acid metabolism and the production of bioactive compounds [74–76], further supporting its functional importance within the gut ecosystem. Additionally, *W. cibaria* was enriched in glycoside hydrolase (GH) and glycosyltransferase (GT) families. The co-enrichment of these enzyme families indicates that *W. cibaria* may contribute to both the breakdown of dietary carbohydrates and the transformation of carbohydrate-derived intermediates [77, 78], thereby influencing rumen fermentation capacity. Consistent with metagenomic results, *W. cibaria* contributes significantly to CAZyme activity, particularly through the enrichment of GT families, suggesting an active role in carbohydrate transformation processes. These carbohydrate-active functions may provide energy and metabolic intermediates that further support amino acid biosynthesis and microbial metabolic interactions. Collectively, these findings highlight *Weissella* as a key functional mediator linking microbial community shifts to metabolic production.

Importantly, integrative multi-omics analysis revealed a coherent microbiota–metabolite–host metabolic axis. Correlation analysis demonstrated that rumen-derived metabolites, including serine, threonine, choline, and hypoxanthine, were positively associated with muscle glutamate levels and the expression of key metabolic genes such as *BCAT2* and *IDH1*/*IDH3A*. These findings suggest that rumen metabolites may act as critical mediators linking microbial activity to host metabolism. Furthermore, mediation analysis provided additional evidence for potential causal relationships, showing that these metabolites statistically mediated the association between *Weissella* and muscle glutamate metabolism. It has previously been reported that microbial metabolites act as mediators of microbiota–host interactions and regulate host transcriptional and metabolic pathways [79, 80]. This further highlights a metabolite-dependent regulatory mechanism, in which microbial-derived metabolites act as key mediators shaping host physiological responses.

Collectively, the present study demonstrates that dietary PSE supplementation is associated with alterations in rumen microbial composition and metabolic profiles, accompanied by changes in muscle amino acid and energy metabolism. Glutamate-related metabolism represented an important metabolic characteristic associated with PSE supplementation, potentially linking rumen-derived metabolic changes with host muscle metabolic responses. These findings provide new insights into the potential microbiota–metabolite–host metabolic relationships involved in nutritional regulation of meat quality in ruminants. Furthermore, the identification of *Weissella* as a candidate microbial taxon provides a foundation for future investigations using microbial isolation, functional characterization, and intervention approaches to clarify its specific contribution to host metabolism and meat quality formation.

## Conclusion

Dietary supplementation with PSE improved meat quality in Hainan black goats, as evidenced by higher ultimate pH, enhanced color stability, and improved water-holding capacity, without affecting growth performance or carcass characteristics. PSE supplementation was associated with alterations in rumen microbial composition, including enrichment in the relative abundance of genera such as *Weissella* and *Ruminococcus*, as well as shifts in rumen metabolic profiles. These microbial and metabolic changes were accompanied by increased levels of metabolites related to amino acid metabolism, including serine, threonine, choline, and hypoxanthine. Multi-omics integration and mediation analysis revealed statistical associations among *Weissella* abundance, rumen metabolites, and muscle glutamate accumulation and metabolic gene expression, supporting a potential microbiota–metabolite–host metabolic relationship. Furthermore, PSE supplementation was associated with enhanced glutamate-related amino acid metabolism and alterations in energy metabolism pathways in muscle, which may contribute to improved postmortem metabolic characteristics.

Overall, this study provides new insights into the association network linking PSE-induced changes in rumen microbial ecology, microbial metabolites, and host muscle metabolism in relation to goat meat quality. These findings highlight the potential of PSE as a nutritional strategy for improving meat quality through coordinated modulation of rumen microbial and host metabolic profiles.

## Supplementary Information

Additional file 1: Table S1. Compositions and nutrient levels of the experimental basal diet. Table S2. The chemical components of *Piper sarmentosum* Roxb. extract. Table S3. Differential muscle metabolites between CON and 600PSE groups. Table S4. Differential muscle genes between CON and 600PSE groups. Table S5. Differential rumen metabolites between CON and 600PSE groups. Table S6. Differential ruminal microbes between CON and 600PSE groups based on LEfSe analysis. Table S7. Source tracing analysis of rumen differential metabolites between CON and 600PSE groups.

Additional file 2: Table S8. Quantitative Real-time polymerase chain reaction system. Table S9. Gene primer sequences for qPCR used to validate RNA-seq. Fig. S1. The qPCR validation of key differentially expressed genes in CON and 600PSE groups.

Additional file 3: Fig. S2. A Number of positive and negative charged ion metabolites identified. B Classification of identified metabolites. C Statistical chart of KEGG enrichment counts for differentially metabolites. D Targeted metabolomic analysis of glutamic acid between CON and 600PSE groups.

Additional file 4: Fig. S3. A Metagenome α diversity indices of Sob, Shannon, and Simpson between the CON and 600PSE groups. B The PCoA of metagenome β diversity between the CON and 600PSE groups.

Additional file 5: Fig. S4. Differentially regulated metabolic pathway in glycine, serine, and threonine metabolism.

## Data Availability

The rumen metagenomic sequencing data generated in this study have been uploaded in the NCBI Sequence Read Archive (SRA) under accession number PRJNA1455142. The raw reads of the muscle transcriptome sequencing data have been uploaded in the NCBI SRA under accession number PRJNA1455417.

## Abbreviations

ADG: Average daily gain
ADFI: Average daily feed intake
ANOSIM: Analysis of Similarities
BCAT2: Branched-chain amino acid transaminase 2
CP: Crude protein
DMI: Dry matter intake
DMs: Differentially expressed metabolites
DEGs: Differentially expressed genes
EE: Ether extract
F/G: Feed-to-gain ratio
GLUL: Glutamine synthetase
GSEA: Gene set enrichment analysis
IDH1: Isocitrate dehydrogenase (NADP(+)) 1
IDH3A: Isocitrate dehydrogenase 3 (NAD(+)) alpha
LEfSe: Linear discriminant analysis Effect Size
MAG: Metagenome-assembled genome
NES: Normalized enrichment score
OPLS-DA: Orthogonal partial least squares discriminant analysis
PCoA: Principal coordinates analysis
PLS-DA: Partial least squares discriminant analysis
PSE: *Piper sarmentosum* Roxb. extract
qPCR: Quantitative real-time polymerase chain reaction
TCA cycle: Citrate cycle
