# Supplementary material for: Multi-omics reveals that dietary Piper sarmentosum Roxb. extract improves goat meat quality via the microbiota–muscle amino acid metabolic axis
Source: J Anim Sci Biotechnol. 2026 Oct 13;17:194. doi: 10.1186/s40104-026-01509-5 (PMC13660090; doi:10.1186/s40104-026-01509-5)
Supplement: Supplementary file 1 [file 40104_2026_1509_MOESM3_ESM.docx]

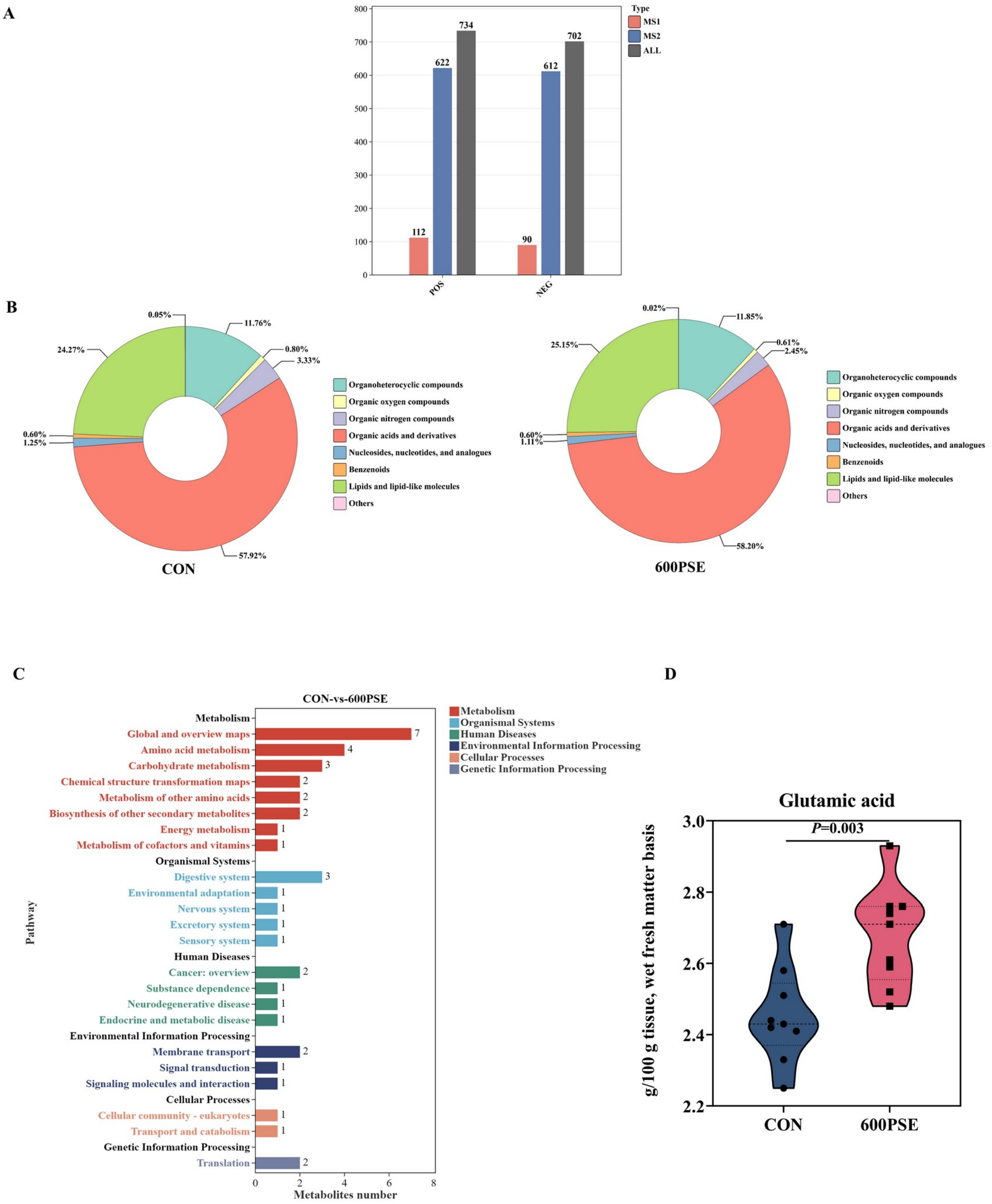
A
Type
MS1
MS2
ALL
112
622
734
90
612
702
POS
NEG
B
Organoheterocyclic compounds
Organic oxygen compounds
Organic nitrogen compounds
Organic acids and derivatives
Nucleosides, nucleotides, and analogues
Benzenoids
Lipids and lipid-like molecules
Others
0.05%
11.76%
0.80%
3.33%
24.27%
0.60%
1.25%
57.92%
CON
0.02%
11.85%
0.61%
2.45%
25.15%
0.60%
1.11%
58.20%
600PSE
C
CON-vs-600PSE
Metabolism
Organismal Systems
Human Diseases
Environmental Information Processing
Cellular Processes
Genetic Information Processing
Global and overview maps 7
Amino acid metabolism 4
Carbohydrate metabolism 3
Chemical structure transformation maps 2
Metabolism of other amino acids 2
Biosynthesis of other secondary metabolites 2
Energy metabolism 1
Metabolism of cofactors and vitamins 1
Digestive system 3
Environmental adaptation 1
Nervous system 1
Excretory system 1
Sensory system 1
Cancer: overview 2
Substance dependence 1
Neurodegenerative disease 1
Endocrine and metabolic disease 1
Membrane transport 2
Signal transduction 1
Signaling molecules and interaction 1
Cellular community - eukaryotes 1
Transport and catabolism 1
Translation 2
Pathway
Metabolites number
D
Glutamic acid
P=0.003
g/100 g tissue, wet fresh matter basis
CON
600PSE
