# Supplementary material for: Multi-omics reveals that dietary Piper sarmentosum Roxb. extract improves goat meat quality via the microbiota–muscle amino acid metabolic axis
Source: J Anim Sci Biotechnol. 2026 Oct 13;17:194. doi: 10.1186/s40104-026-01509-5 (PMC13660090; doi:10.1186/s40104-026-01509-5)
Supplement: Supplementary file 1 [file 40104_2026_1509_MOESM2_ESM.docx]

**qRT-PCR validation of transcriptome sequencing data**

To validate the transcriptome data, quantitative real-time polymerase chain reaction (qRT-PCR) was performed on key differentially expressed genes (*GLUL*, *IDH1*, *CAMK2A*, *PDXK*, *FADS1*, *SCD*, and *NRG4*) identified in the RNA-seq analysis. qRT-PCR was carried out using the SYBR Green I dye method on an Applied Biosystems QuantStudio™ 5 real-time quantitative PCR instrument (Thermo Fisher Scientific, USA). The reactions were prepared using the PerfectStart® Green qPCR SuperMix (+Universal Passive Reference Dye) kit (TransGen Biotech Co., Ltd., Beijing, China), following the manufacturer’s instructions (Table 1). Primers (Table 2) were designed based on goat (*Capra hircus*) gene sequences available in the NCBI database using the Primer-BLAST online tool (https://www.ncbi.nlm.nih.gov/tools/primer-blast/) and synthesized by Sangon Biotech Co., Ltd. (Shanghai, China). The relative expression levels of the target genes were calculated using the 2^⁻ΔΔCt^ method.

Table S8 Quantitative Real-time polymerase chain reaction system

| Component | Volume |
| --- | --- |
| cDNA | 1 ul |
| Forward Primer（10 uM） | 0.6 ul |
| Reverse Primer（10 uM） | 0.6 ul |
| 2x*PerfectStart* Green qPCR SuperMix | 10 ul |
| Nuclease-free Water | 7.8 ul |
| Total volume | 20 ul |

Table S9 Gene primer sequences for qRT-PCR used to validate RNA-seq

| Gene |  | Sequence（forward/reverse） |
| --- | --- | --- |
| *18S rRNA* | Forward primer | GTGGTGTTGAGGAAAGCAGACA |
|  | Reverse primer | TGATCACACGTTCCACCTCATC |
| *IDH1* | Forward primer | GGACAAAAGCCGGGAAGAGA |
|  | Reverse primer | GACTCAGCTTACCTCCTGCG |
| *GLUL* | Forward primer | GGAGAAGAGCCAGCTGTCTAAG |
|  | Reverse primer | CCACTCGGGCAACTCTTCTA |
| *NRG4* | Forward primer | CAGTGTCCACCACGGAACTAC |
|  | Reverse primer | GTTGTGCGTGTCCTTAGCAG |
| *CAMK2A* | Forward primer | AAAGAAAGTCCAGTTCCAGCG |
|  | Reverse primer | CTCTTGTTTCCGCACTTTGGT |
| *pdxK* | Forward primer | GCGGTGAACTCTGTCCAGTT |
|  | Reverse primer | CATCACGGGGTCACACACATA |
| *SCD* | Forward primer | GTGATGTTCCAGAGGAGGTACT |
|  | Reverse primer | GGGGTTGATGGTCTTGTCGT |
| *FADS1* | Forward primer | TGGCTCGTGATTGACCGAAA |
|  | Reverse primer | CCACAAAAGGATCCGTGGCA |

Figure S1 qRT-PCR validation of key differentially expressed genes in CON and 600PSE groups
