# Supplementary material for: Multi-omics reveals that dietary Piper sarmentosum Roxb. extract improves goat meat quality via the microbiota–muscle amino acid metabolic axis
Source: J Anim Sci Biotechnol. 2026 Oct 13;17:194. doi: 10.1186/s40104-026-01509-5 (PMC13660090; doi:10.1186/s40104-026-01509-5)
Supplement: Supplementary file 3 [file 40104_2026_1509_MOESM4_ESM.docx]

Figure S3. A. Metagenome α diversity indices of Sob, Shannon, and Simpson between the CON and 600PSE groups. B. The PCoA of metagenome β diversity between the CON and 600PSE groups.
