# Supplementary material for: Multi-omics reveals that dietary Piper sarmentosum Roxb. extract improves goat meat quality via the microbiota–muscle amino acid metabolic axis
Source: J Anim Sci Biotechnol. 2026 Oct 13;17:194. doi: 10.1186/s40104-026-01509-5 (PMC13660090; doi:10.1186/s40104-026-01509-5)
Supplement: Supplementary file 4 [file 40104_2026_1509_MOESM5_ESM.docx]

Figure S4 Differentially regulated metabolic pathway in glycine, serine, and threonine metabolism
